# Recent Progress on Asymmetric Carbon- and Silica-Based Nanomaterials: From Synthetic Strategies to Their Applications

**DOI:** 10.1007/s40820-021-00789-y

**Published:** 2022-01-17

**Authors:** Haitao Li, Liang Chen, Xiaomin Li, Daoguang Sun, Haijiao Zhang

**Affiliations:** 1 Institute of Nanochemistry and Nanobiology, Shanghai University, Shanghai, 200444 People’s Republic of China; 2 Department of Chemistry, Laboratory of Advanced Nanomaterials, Shanghai Key Laboratory of Molecular Catalysis and Innovative Nanomaterials, State Key Laboratory of Molecular Engineering of Polymers, Collaborative Innovation Center of Chemistry for Energy Nanomaterials (2011-iChEM), Fudan University, Shanghai, 200433 People’s Republic of China

**Keywords:** Carbon- and silica-based nanoparticles, Asymmetric structure, Synthetic strategies, Energy storage and conversion, Biomedicine

## Abstract

**Highlights:**

- The synthetic strategies and fundamental mechanisms of various asymmetric carbon- and silica-based nanomaterials were systematically summarized.
- The advantages of asymmetric structure on their related applications were clarified by some representative applications of asymmetric carbon- and silica-based nanomaterials.
- The future development prospects and challenges of asymmetric carbon- and silica-based nanomaterials were proposed.

**Abstract:**

Carbon- and silica-based nanomaterials possess a set of merits including large surface area, good structural stability, diversified morphology, adjustable structure, and biocompatibility. These outstanding features make them widely applied in different fields. However, limited by the surface free energy effect, the current studies mainly focus on the symmetric structures, such as nanospheres, nanoflowers, nanowires, nanosheets, and core–shell structured composites. By comparison, the asymmetric structure with ingenious adjustability not only exhibits a larger effective surface area accompanied with more active sites, but also enables each component to work independently or corporately to harness their own merits, thus showing the unusual performances in some specific applications. The current review mainly focuses on the recent progress of design principles and synthesis methods of asymmetric carbon- and silica-based nanomaterials, and their applications in energy storage, catalysis, and biomedicine. Particularly, we provide some deep insights into their unique advantages in related fields from the perspective of materials’ structure–performance relationship. Furthermore, the challenges and development prospects on the synthesis and applications of asymmetric carbon- and silica-based nanomaterials are also presented and highlighted.
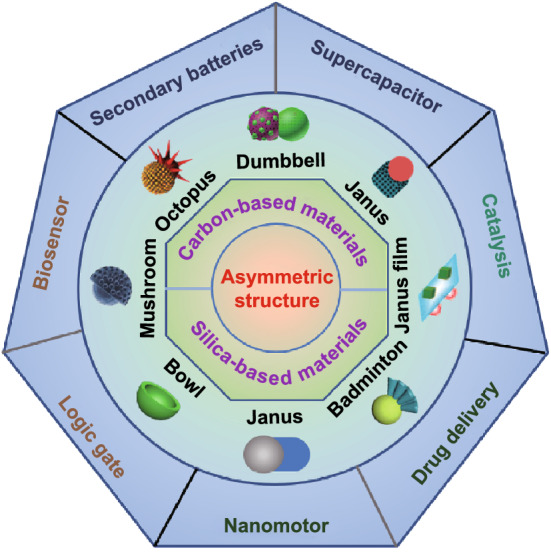

## Introduction

As two of the most important branches in material science, carbon- and silica-based nanomaterials have shown fascinating application prospects in energy, catalysis, adsorption, and biology fields due to their outstanding advantages such as structural adjustability, excellent biocompatibility, and easy to functionalize [1–6]. Over the past few decades, carbon- and silica-based nanomaterials with all kinds of structures have been well developed, and their structure–performance relationship has also been deeply explored to a certain extent [3, 7–9]. Despite some of the achievements made, most of them were mainly synthesized and employed in the form of conventional symmetric structures, e.g., uniform nanospheres and some core–shell nanocomposites.

**Fig. 1:**
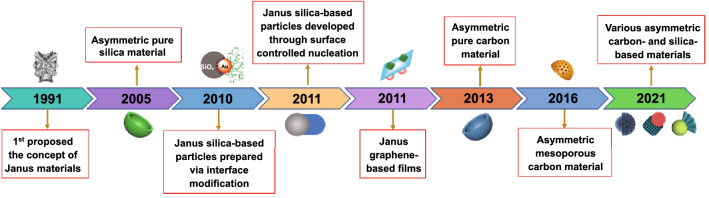
Historical development of asymmetric carbon- and silica-based nanomaterials

**Fig. 2:**
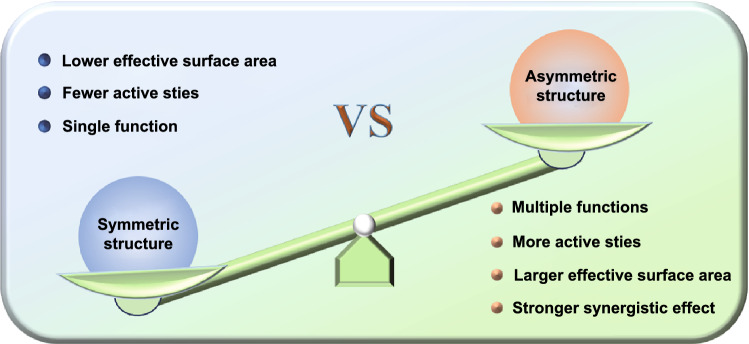
Comparisons of advantages and disadvantages of symmetric and asymmetrical structures

Since Gennes et al. proposed the concept of Janus in 1991 for the first time [10], the asymmetric structure has gradually attracted increasing attention. In the past more than two decades, different asymmetric nanomaterials have been massively designed, and the definition of asymmetric structure was far beyond the original Janus structure (Fig. 1). It includes not only the Janus structure with different surface properties, but also the asymmetry in topography, such as bowl-shaped [11], snowman-shaped [12], disk-shaped [13], and raspberry-shaped structures [14]. Compared with the conventional symmetric structures, asymmetric structures possess some unique advantages. As shown in Fig. 2, multiple functions are one of the main advantages of asymmetric structures. Due to the different surface physicochemical properties or different components, asymmetric nanoparticles can contain several distinct properties simultaneously, such as hydrophilicity and hydrophobicity, optical, and magnetic properties. Thus, it is an ideal choice for designing “nano-intelligent systems” based on a single asymmetric particle and shows a great application potential in the fields of electrochemistry, interfacial stabilizer, and biomedicine. The stronger synergistic effect is another advantage of asymmetric structure. The distinct domains of asymmetric structure can work independently without interfering with each other and even can cooperate with each other to realize significantly improved properties in comparison with traditional core–shell symmetric structures. On the other hand, the asymmetric structure with ingenious adjustability exhibits a larger effective surface area accompanied with more active sites, which is critical to the properties of the material. For example, when used as the electrode materials, the larger effective specific surface area can maximize the electrolyte permeation and enhance the contact area between electrode and electrolyte interfaces, which can effectively boost the electrochemical performances. Owing to those attractive features of asymmetric structure, the development of carbon- and silica-based asymmetric nanomaterials has flourished in the past ten years. With the exploration of advanced synthetic methods, the carbon and silica can be married with diversified functional nanomaterials in the form of asymmetric structure, thereby significantly extending their application fields.

Although several previous reviews have demonstrated the design and fabrication of asymmetric nanomaterials [15–17], the underlying synthetic mechanism still needs to be discussed in detail. Besides, the systematic reviews on the asymmetric carbon- and silica-based nanomaterials have rarely been reported. Moreover, the advantages of asymmetric structured carbon- and silica-based nanomaterials in specific fields should be elaborated from the perspective of structure–performance relationship. Therefore, it is urgent to summarize the preparation methods and design mechanisms of asymmetric carbon- and silica- materials, as well as the relationship between their structure and properties to better understand and widely apply the kind of promising nanomaterials.

Here, we mainly focus on the lasted developments of asymmetric carbon- and silica-based nanomaterials in this review. First, the synthetic strategies and fundamental mechanisms for various asymmetric carbon- and silica-based nanomaterials are systematically summarized. Then, some representative applications of asymmetric carbon- and silica-based nanomaterials are elaborated to clarify the advantages of asymmetric structure on their performance. At last, the future development prospects of asymmetric structure carbon- and silica-based nanomaterials have been also proposed and discussed.

## Design Principles and Synthesis Strategies of Asymmetric Carbon- and Silica-Based Nanomaterials

As mentioned above, a diversity of carbon- and silica-based asymmetric nanomaterials have been developed in recent years with the rapid development of nanotechnology. However, the synthesis of asymmetric carbon nanomaterials differs slightly from the asymmetric silica nanomaterials. In general, carbon nanomaterials can be obtained by the carbonization of polymer-based nanomaterials, resulting in the formation of geometrically asymmetric structures. With regard to asymmetric silica nanomaterials, the well-developed “silica chemistry” greatly facilitates the preparation of asymmetric silica nanomaterials, realizing pure silica nanostructures with geometrical asymmetry or nanocomposites with two or more districts that differ in chemical compositions or properties. In this section, the design principles and synthesis strategies will be scientifically discussed according to the compositions of asymmetric nanomaterials, ranging from pure carbon and silica to their composite counterparts.

### Asymmetric Pure Carbon Nanoparticles

Different geometrically asymmetric carbon nanoparticles, such as bowl-, red blood cell (RBC)-, and vase-shaped structures, have been reported. The synthesis methods of asymmetric carbon nanomaterials are similar to those traditional symmetric nanomaterials, which mainly include hard-templating, soft-templating, self-templating techniques [11, 18–26]. Nevertheless, their formation mechanisms are essentially different. Here, the mainly synthesis strategies and fundamental mechanisms for asymmetric carbon nanoparticles will be presented.

**Fig. 3:**
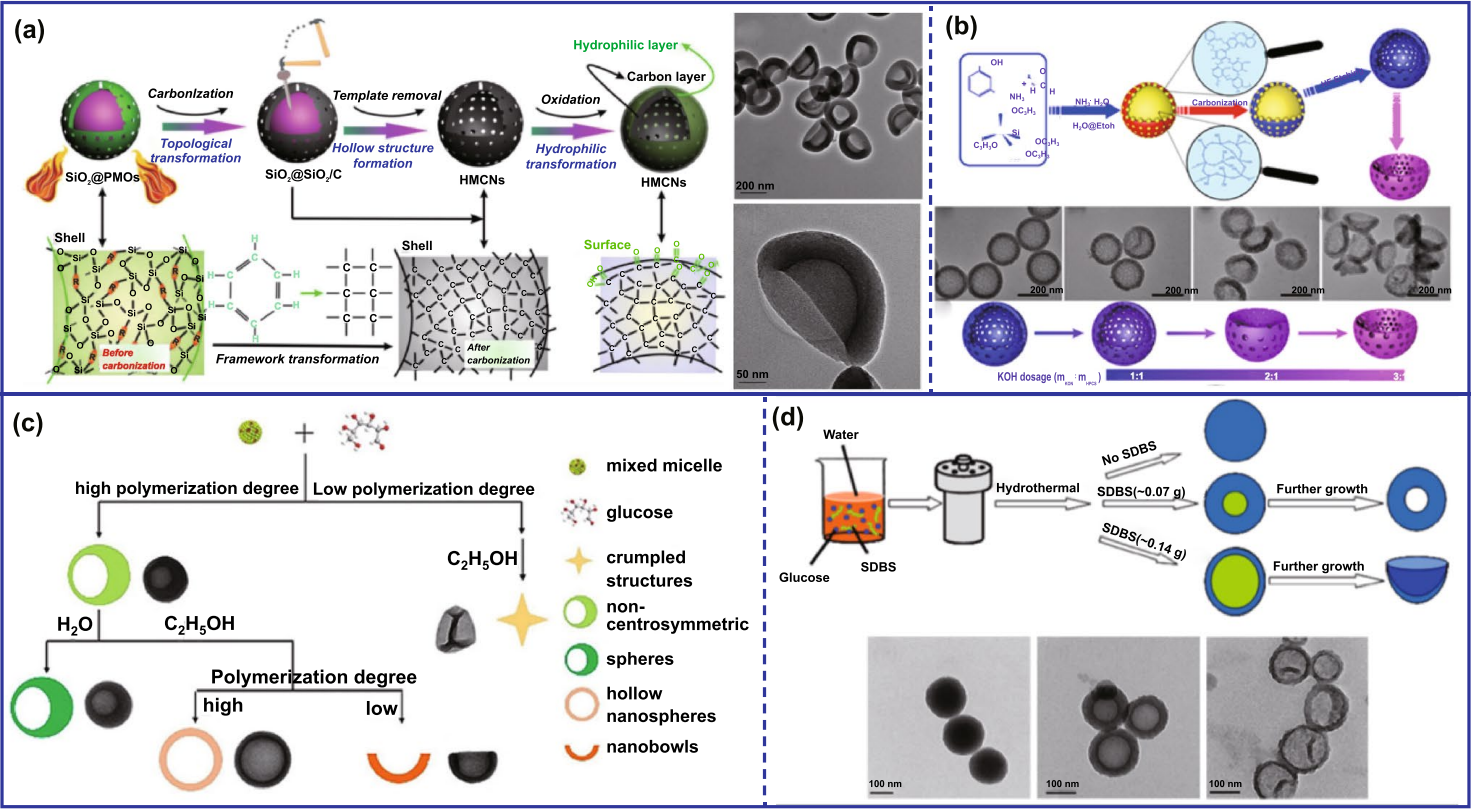
Various synthetic strategies for preparing asymmetric pure carbon nanoparticles. **a** Alkali etching destroys the rigid structure to obtain a bowl-shaped carbon material. Reproduced with permission from Ref. [27]. Copyright 2013, Wiley–VCH. **b** Preparation of asymmetric carbon nanoparticles by subsequent activation pore-making. Reproduced with permission from Ref. [32]. Copyright 2018, American Chemical Society. **c** Selectively dissolving oligomers by solvents to obtain bowl-shaped carbon nanoparticles. Reproduced with permission from Ref. [33]. Copyright 2018, Wiley–VCH. **d** Preparation of asymmetric carbon nanoparticles by adjusting the thickness of the precursor. Reproduced with permission from Ref. [20]. Copyright 2015, Elsevier B.V

The low-surface-rigidity-induced deformation of the hollow sphere is the most facile and fundamental mechanism to prepare asymmetric carbon nanoparticles. The rigidity of the hollow polymer sphere can be precisely adjusted by various means. Selective removal of nonessential components from sphere shells is one of the most common methods to alter the rigid structure of nanoparticles. Shi’s group prepared RBC-like carbon nanoparticles by using SiO_2_ as the hard template and mesoporous organosilica as the carbon and silica precursor, respectively (Fig. 3a). In this case, the SiO_2_ core was preferentially etched and the hollow SiO_2_/C nanospheres were obtained after carbonization. After further removing the SiO_2_ in shell, the rigidity of remaining carbon shell was not enough to support the original hollow structure, thus giving rise to the unique RBC-shaped carbon nanoparticles [27]. By using similar strategy, a series of bowl- and RBC-shaped carbon nanoparticles have been prepared [19, 22, 27–30]. Evenly, the bowl-like mesoporous carbon can also be prepared [31]. However, the above synthesis processes are usually complicated and uncontrollable. Recently, activation pore-making strategy was used to adjust the rigid structure of the original materials (Fig. 3c) [32]. In this strategy, the hollow carbon spheres were first prepared by a typical one-pot in situ template route. Then, the obtained hollow carbon spheres were mixed with different dosages of KOH for high-temperature activation pore-making. In this process, low KOH dosage can etch the shell to enhance the porosity of the spherical shell, while under high KOH dosage, the seriously etched shell would collapse due to inward depression to form a bowl-like morphology. Notably, this synthetic strategy showed a higher application value. Besides, some organic solvents also can be used to remove the nonessential components of polymer sphere. For example, ethanol was skillfully selected as organic solvent to remove incomplete oligomers from polymer spheres and prepared bowl-shaped carbon nanoparticles after carbonization (Fig. 3b) [33]. Specifically, the hollow polymer nanospheres were first prepared by the hydrothermal carbonization of glucose monomers using poly(ethylene glycol) (P123) and sodium dodecyl sulfate (SDS) as soft templates. Then, the oligomer remained in the polymer shell can be preferentially dissolved by ethanol, which leads to the decreased rigidity of the nanospheres, and finally induced the buckling of the shells to form the bowl-like morphology.

In addition, the surface rigidity of hollow polymer sphere can be adjusted by controlling the thickness of polymer shell during synthesis. For example, our group prepared carbon nanoparticles with diverse structures by using P123 and sodium dodecylbenzene sulfonate (SDBS) as the soft templates and glucose as the carbon source (Fig. 3d) [20]. It was found that the SDBS played a crucial role on the morphology of prepared carbon nanospheres. Specifically, the structure of carbon nanoparticles changed from solid to hollow sphere when the SDBS was added. And the shell thickness of the hollow carbon sphere was significantly reduced as the amount of SDBS further increased. When the thickness was reduced to a certain extent, the rigidity of the carbon shell cannot support the original spherical structure. Finally, the bowl-shaped structure was formed. Moreover, other synthesis parameters, such as reaction temperature and precursor concentration, also can adjust the thickness of the polymer shell. Zhang and co-workers systematically studied the influence of synthetic parameters on the structure of carbon nanoparticles in a recent work, in which the mixed micelles P123 and sodium oleate (SO) were used as soft templates and glucose was used as carbon source [34]. They found that the concentration of precursor, surfactant, and reaction time had a decisive effect on the thickness of the carbon shell. Thus, the structure can be changed from sphere to bowl-like by adjusting the above synthetic parameters. Evenly, the open structured carbon spheres can be fabricated when the polymer shells were cracked. For example, open structured carbon nanoparticles were fabricated using phenolic resin (PR) as precursor and polystyrene (PS) spheres as template [35]. The inner pressure of the hollow PR shell was gradually increased as the decomposition of PS core at high temperature carbonization. Accordingly, the carbon shells were cracked and the open structured carbon spheres were formed once the pressure exceeded the endurance of the carbon shell. Analogously, Lou's group also fabricated single-hole cobalt-/N-doped carbon (Co/NC) hollow particles by using PS spheres as template [36]. Although the above strategies can simply prepare various asymmetric carbon nanoparticles, most of them were mainly bowl-like structures, while mesoporous asymmetric carbon materials are difficult to be well prepared.

**Fig. 4:**
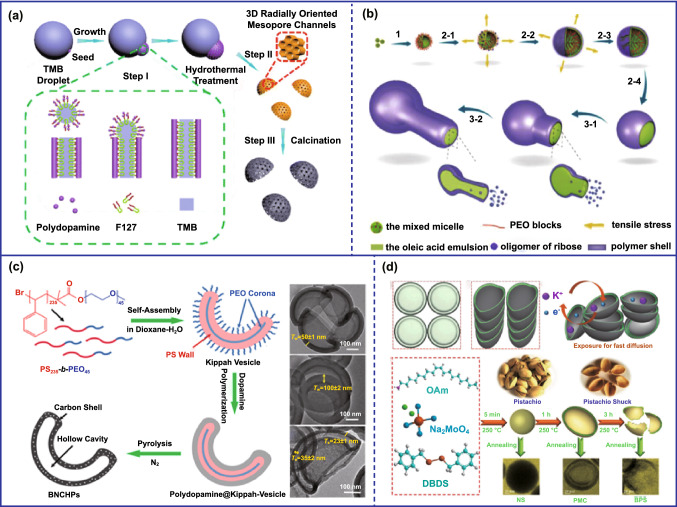
**a** Fabrication of asymmetric mesoporous carbon nanoparticles through interfacial assistance. Reproduced with permission from Ref. [37]. Copyright 2016, American Chemical Society. **b** Asymmetric flask-like carbon nanoparticles were fabricated using the high-temperature hydrothermal approach at the emulsion interface. Reproduced with permission from Ref. [40]. Copyright 2017, American Chemical Society. **c** Fabrication of bowl-like structures through the asymmetric template. Reproduced with permission from Ref. [21]. Copyright 2016, Royal Society of Chemistry. **d** Fabrication of asymmetric carbon through other method. Reproduced with permission from Ref. [43]. Copyright 2018, Wiley-VCH

The interfacial assembly, for example emulsion interface, provides an effective way for designing the asymmetric carbon nanoparticles. Most importantly, some mesoporous or more interesting asymmetric structure can be achieved based on the facile strategy. For instance, asymmetric mesoporous carbon nanoparticles were fabricated by using poly(propylene glycol)-block-poly(ethylene glycol)-block-poly(propylene glycol) (F127) and 1,3,5-trimethylbenzene (TMB) mixed emulsion as soft template and dopamine hydrochloride as carbon precursor. Specifically, the oil–water interface of emulsion droplet formed by F127/TMB/polydopamine can direct the island nucleation and anisotropic growth of polydopamine nanoparticles. With the increased amount of TMB, the morphology of obtained polydopamine nanoparticles can evolve from large-pore mesoporous structure to bowl-like nanoparticles with radial meso-channel (Fig. 4a) [37]. However, the assembly mechanism of the above asymmetric mesoporous carbon lacked in-depth research. Recently, Mai’s group prepared mushroom-shaped mesoporous carbon nanoparticles and investigated the assembly mechanism through the interfacial interactions [38]. They found that the average size of oil droplets has a significant influence on the morphology of the consequent carbon nanoparticles. When the size of oil droplets was relatively small, the mushroom-shaped mesoporous carbon nanoparticles were obtained under the action of interfacial tension. The structure of carbon nanoparticles changed from wheel-shaped to acorn-like as the size of oil droplets further increased. Moreover, Lee et al. designed mesoporous carbon bowls based on the polymer blend-directed anisotropic self-assembly strategy [39]. The spreading coefficients (*λijk*) in poly(ethylene oxide)-*b*-poly(styrene) (BCP), homopoly(methylmethacrylate) (hPMMA), and homopoly(styrene) (hPS) ternary immiscible blends were introduced to understand the assembly behavior. According to the Harkin’s theory, the tendency for BCP phase to spread over hPS was slightly more favored due to the high value of *λ*_PMMA/BCP/PS_, leading to bowl-like mesoporous structures. In addition, the as-made products were microtomed at a thickness of 100 nm for transmission electron microscopy to elucidate the morphology formation and further justified the phase behaviors of blends. However, the precursors used in the above methods were usually limited to those that easily polymerized at low temperatures due to the weak interaction between emulsion templates and precursors at high temperature. Recently, asymmetric flask-like carbon nanoparticles were fabricated using the high-temperature hydrothermal approach at the emulsion interface formed by mixing micelles of P123 and SO (Fig. 4b) [40]. Here, the ribose was first polymerized at the emulsion interface formed by mixing micelles of P123 and SO as the temperature increased. Meanwhile, the hydration level of PEO block in P123 decreased and penetrated into SO; thereby, the tensile stress generated by volume expansion of nano-emulsions and finally caused the polymer shell cracked when the pressure exceeded the critical pressure. As the reaction progressed, the nano-emulsion template flowed out to form a fresh template surface on which the precursors would continue to be polymerized, causing the asymmetric flask-like hollow carbonaceous nanoparticles. Moreover, the length of the neck and the diameter of the inner cavity can also be adjusted by controlling the reaction time. In addition, the interactions between the templates and precursors can also be used to regulate the structure during the preparation of hollow carbon spheres using hard templates. Stucky's group prepared concave-structured carbon nanoparticles by regulating the interface adhesion between template core and carbon shell. They used polystyrene@carbon (PS@C) nanoparticles to prove a concept of “swelling-induced plastic deformation.” The adhesive force between the PS core and the carbon shell was completely changed by a heating–cooling treatment. Concretely, the PS core was significantly swelled under the alcohol thermal treatment, which rapidly generates high tensile stress to induce the elastic expansion of the carbon shell. After cooling, the shrinkage of PS core turned to pull the enlarged shell under the effect of the adhesive force, thereby resulting in buckling of the carbon shells to form the concave-structured carbon nanoparticles [41]. Later, Zhao and co-workers coated a layer of silica on the surface of the polymer nanospheres to form a polymer–silica interface. Similarly, the steam repulsion, interface tension, and constriction force were generated at high temperature carbonization. Thus, the polymer precursor would be shrunk asymmetrically under the combined effect of the above force, and the hemispherical carbon nanoparticles were produced after further removing the silica shell [42].

Directly coating carbon precursors onto an asymmetric template or selectively coating on a symmetric template is another available route for fabricating asymmetric carbon structures [44]. For example, bowl-shaped carbon nanoparticles were directly prepared by using kippah-vesicle-shaped polystyrene-block-poly(ethylene oxide) (PS-*b*-PEO) as asymmetric template (Fig. 4c) [21]. And carbon nanocups also were prepared by selectively coated carbon precursor onto ZnO nanorods through chemical vapor deposition [45].

Apart from the above-mentioned synthesis strategies, asymmetric carbon nanoparticles can also be directly synthesized. For example, bowl-shaped carbon nanoparticles were directly prepared by processing worm shells [46]. And Guo's group prepared pistachio-shuck-like MoSe_2_/C nanomaterials by using sodium molybdate, dibenzyl diselenide, and oleylamine as precursors. Meanwhile, the structures can be easily adjusted through controlling the high-temperature treatment time (Fig. 4d) [43].

### Asymmetric Carbon-Based Nanomaterials

Although different asymmetric carbon nanoparticles with unique advantages have been designed, their applications are severely limited because of the individual carbon components. In principle, reasonable design and preparation of asymmetric carbon-based composites can finely adjust their electronic, magnetic, and mechanical properties to extend their applications.

**Fig. 5:**
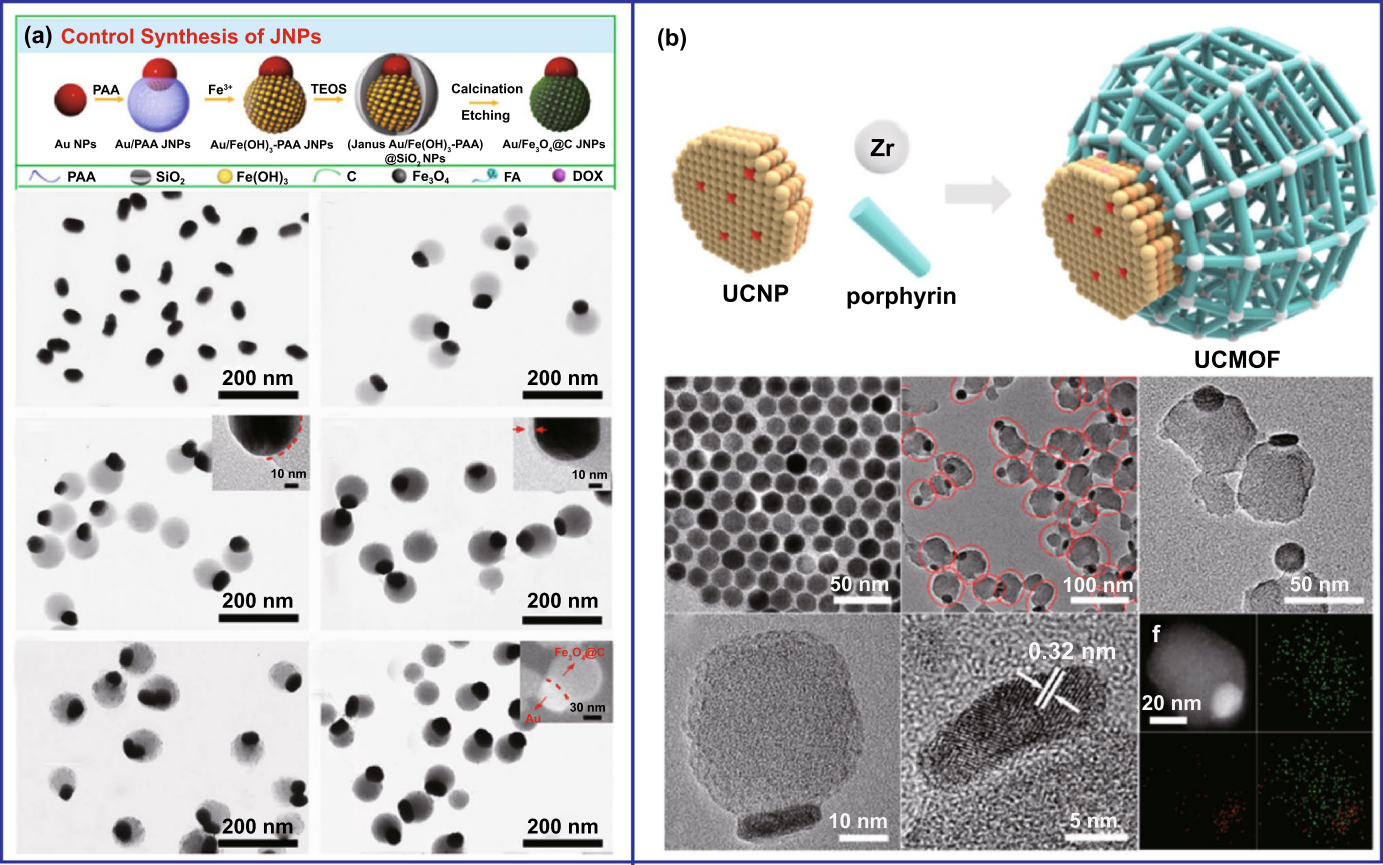
Preparation of various asymmetric polymer- and MOF-based nanoparticles. **a** Preparation of Au/Fe_3_O_4_@C Janus nanoparticles by controlling the surface interfacial energy. Reproduced with permission from Ref. [51]. Copyright 2017, Wiley–VCH. **b** Fabrication of Janus structured MOF-UCNPs by selectively modifying the surface. Reproduced with permission from Ref. [54]. Copyright 2017, American Chemical Society

Generally, it requires the combination of polymer with other functional nanoparticles to realize the carbon-based composites. However, the anisotropic growth of polymer onto different substrates remains a huge challenge. Wang’s group proposed a facile strategy to prepare asymmetric metal–polymer nanocomposites [47]. Interestingly, they found that Au–polyacrylic acid (PAA) Janus nanoparticles (JNPs) were generated by adding the isopropyl alcohol (IPA) into the aqueous solution of citrate–Au NPs and PAA under the alkaline condition. This method was also applicable when the seeds are replaced with other materials (Ag nanocubes [48], gold nanorods [49], Pd nanosheets [50], etc.). Furthermore, more complex asymmetric nanostructures can also be synthesized in a controlled manner by the metal–polymer JNPs as seeds. As an example, the group prepared Au/Fe_3_O_4_@C JNPs by carbonization and etching the Au/Fe(OH)_3_-PAA@SiO_2_ JNPs (Fig. 5a) [51]. The Au–PAA JNPs were first prepared by controlling the surface interfacial energy. Subsequently, Fe^2+^ was selectively deposited on the surface of PAA and hydrolyzed to Fe(OH)_3_. Then, the whole JNPs were coated by SiO_2_ to protect from undesirable agglomeration during the calcination process. Last, the Au/Fe_3_O_4_@C JNPs were obtained by carbonization and etching. Similar to this report, other polymers- and metal organic framework (MOF)-based asymmetric nanoparticles have also been designed [49, 50, 52, 53]. Besides, the asymmetric MOF-based materials have also been synthesized by selectively modifying the surface of seeds. Li’s group prepared Janus-structured MOF-up conversion nanoparticles (UCNPs) by a convenient solvothermal method (Fig. 5b) [54]. In this study, hydrophobic UCNPs were converted into hydrophilic nanocrystals by coating polyvinylpyrrolidone (PVP), and then, the MOF-UCNPs JNPs were obtained through anisotropic growth of MOFs on UCNPs.

**Fig. 6:**
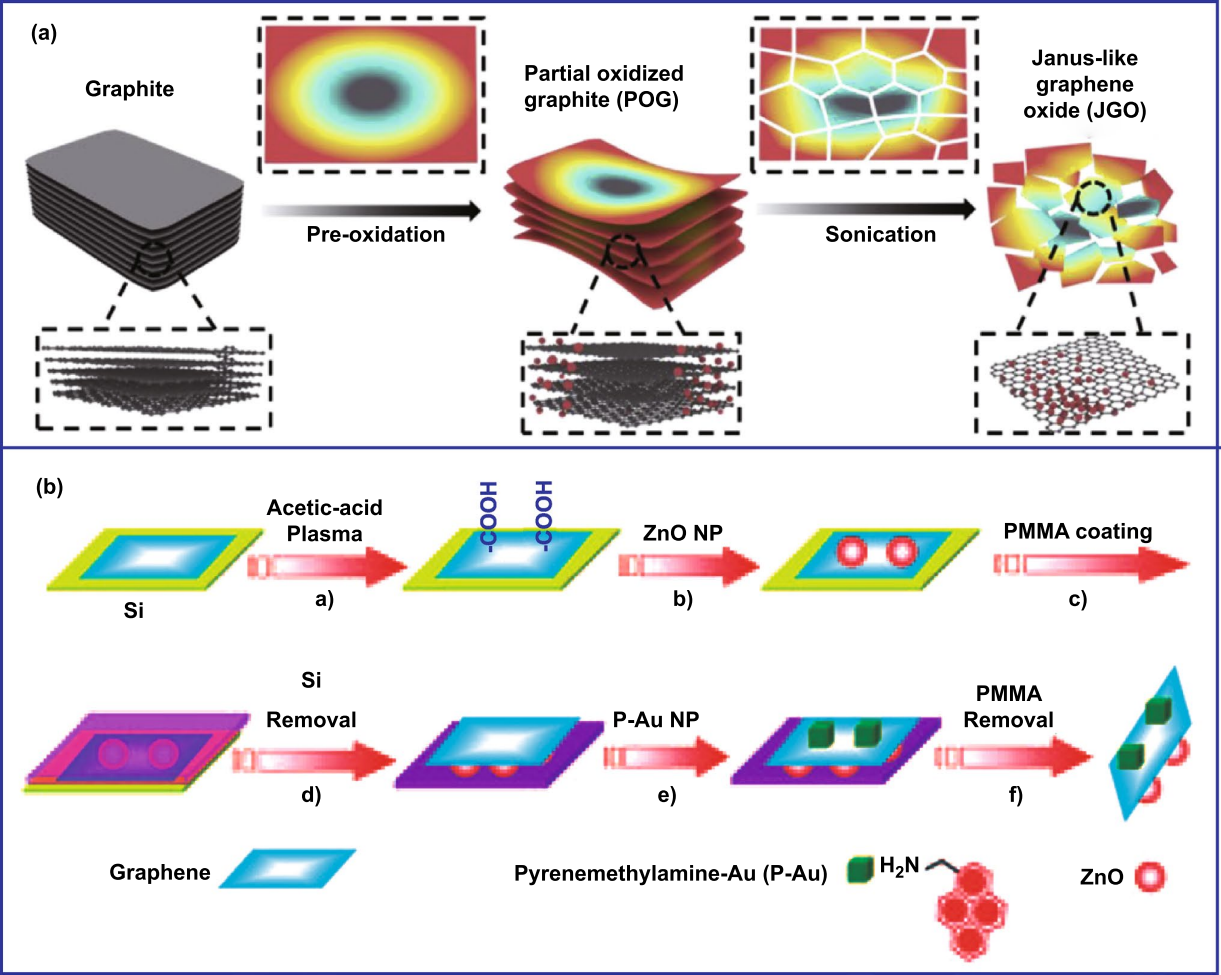
Preparation of various asymmetric carbon-based nanomaterials. **a** Preparation of novel asymmetric structure along the faces of the sheets. Reproduced with permission from Ref. [69]. Copyright 2020, Elsevier B.V. **b** Fabrication of Janus structured ZnO-graphene-Au composite materials by selectively modifying the surface. Reproduced with permission from Ref. [70]. Copyright 2011, Wiley-VCH

Apart from the nanoparticles mentioned above, graphene-based Janus films with heterogeneous layers were another type of promising nanomaterials because of their unique two-dimensional structure, large surface area, and superior performance. The asymmetric graphene-based material was mainly synthesized by selective modification, which involved exposing one face of graphene to atmosphere or solutions while protecting the other side for the preparation of graphene-based Janus structure. According to this template-assisted strategy, a variety of graphene-based Janus films with two distinct functionalized surfaces have been prepared. In 2010, Robinson's group first reported that graphene films grown on Cu foils were fluorinated with xenon difluoride (XeF_2_) gas on one or both sides [55]. After that, diverse graphene-based Janus structural nanomaterials have been designed via different modifying reagents, including small organic molecules [56–58] and polymers [59–62] [63, 64]. Nonetheless, the functionality of above-mentioned Janus graphene nanomaterials was too simple. In 2013, Zhang's group prepared Janus-structured graphene by co-grafting of halogen and aryl/oxygen-functional groups on each side for the first time [65]. In the experiment, they first functionalized one side of graphene by grafting halogen groups and then coated a PMMA film on the single-sided functionalized graphene. Subsequently, the PMMA-coated graphene was peeled off from the substrate and the other side of the graphene was asymmetrically grafted by aryl or oxygen-functional groups. Finally, the Janus graphene was obtained after removing the PMMA film from the graphene. Inspired by this work, Janus graphene functionalized with nitrobenzene diazonium (NBD) and methoxybenzene diazonium (MBD) on the opposite sides of graphene was prepared using aqueous solutions of aryl diazonium molecules [66]. However, the yield obtained was limited by using this method. The above shortcomings can be effectively solved using the Pickering emulsion template [67]. For instance, GO nanosheets were first assembled on the surface of wax particles via a Pickering-type emulsion; then, PMMA was grafted selectively from the exposed face. At last, Janus PMMA-GO-X was collected after removing the wax substrate [68]. Although the synthesis technology of graphene-based Janus structure has matured, the synthesis process was relatively complicated, and the asymmetry was based on opposite sheet faces. Recently, Zhang’s group proposed a modified method to fabricate Janus-like GO (JGO) with a novel asymmetric structure along the faces of the sheets (Fig. 6a) [69]. In this study, the oxidation process preferentially occurs at the edges and defect locations, and the middle part of graphene was protected, which is similar to the inert template protection.

It should be noted that the selective deposition of metals or metal oxides on the surface of graphene can endow graphene with specific electrical and chemical properties. For example, ZnO and Au nanoparticles were successfully deposited on the different sides of graphene (Fig. 6b) [70]. The whole synthetic process was mainly divided into three steps: first, selectively deposit ZnO on the exposed graphene layer; second, deposit a PMMA layer to protect the ZnO and remove the silicon matrix; and finally the other side of the graphene was modified and then gold nanoparticles are deposited. In addition, different metals or metal oxides, such as Au [71, 72], Ag [73, 74], ZnO [75], and Cu_2_O [76], have been selectively deposited on the one side of graphene surface by chemical vapor deposition, electrochemical deposition, and other methods.

### Asymmetric Pure Silica Nanoparticles

Silica nanoparticles are commonly synthesized by the well-developed “sol–gel” chemistry. By regulating the interfacial interaction in reaction system, pure asymmetric silica nanoparticles can be prepared via the wet-chemistry method, but anisotropic nucleation and growth of silica onto different seeds can be realized to prepare hybrid silica-based asymmetric nanoparticles.

**Fig. 7:**
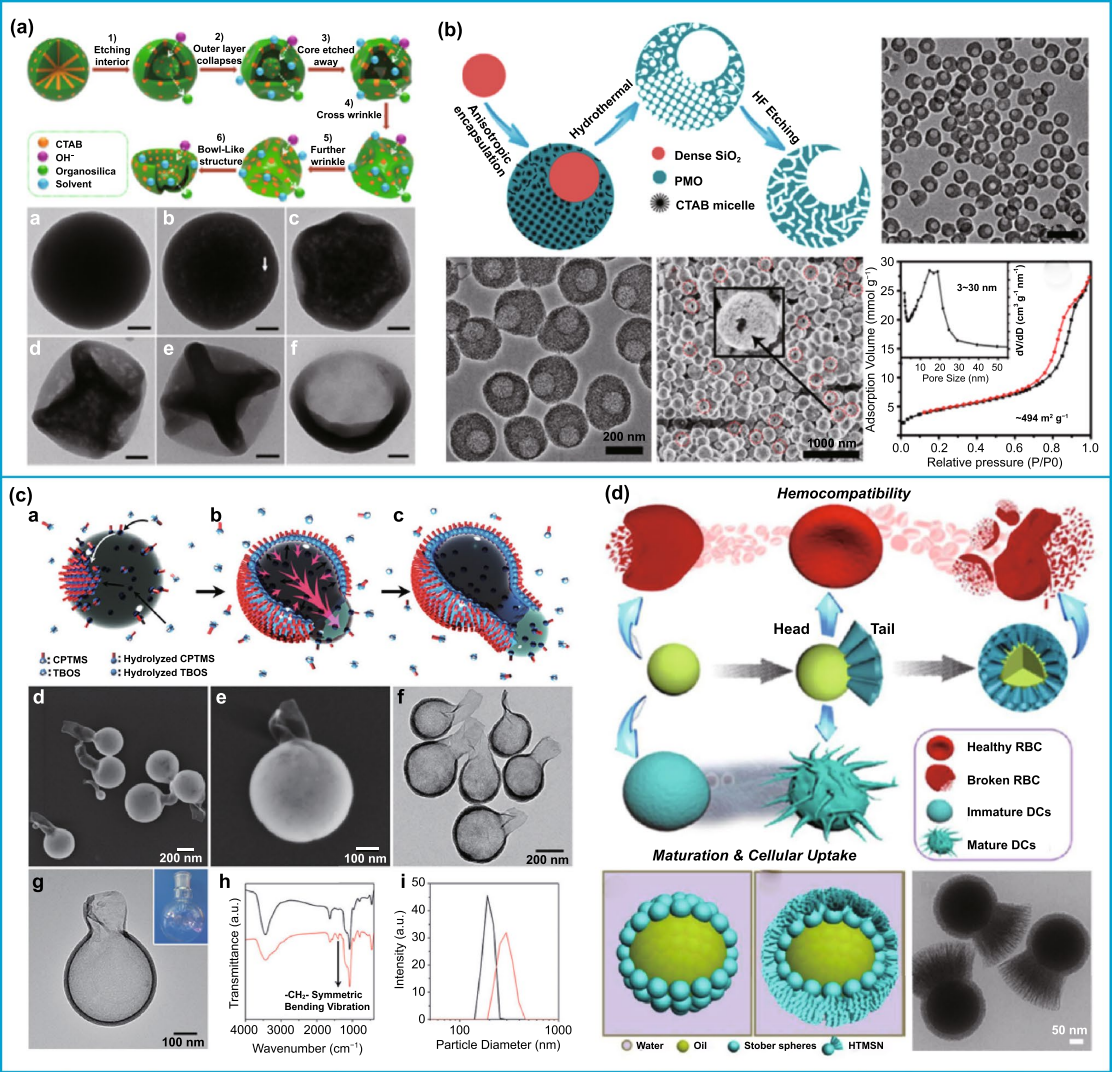
Various synthetic strategies for preparation of asymmetric pure silica nanoparticles. **a** Alkali etching destroys the rigid structure of the silica and results in a bowl-shaped silica material. Reproduced with permission from Ref. [78]. Copyright 2017, American Chemical Society. **b** Fabrication of open-structured silica nanoparticles through chemical etching. Reproduced with permission from Ref. [80]. Copyright 2015, American Chemical Society. **c** Preparation of bottles-like silica nanoparticles through two-phase interface control. Reproduced with permission from Ref. [81]. Copyright 2016, American Chemical Society. **d** Fabrication of badminton-like structure through two-phase interface control. Reproduced with permission from Ref. [82]. Copyright 2017, American Chemical Society

Similarly, asymmetric silica nanoparticles can also be got via changing the rigidity of the silica shell. In 2005, Zoldesi’s group prepared silica capsules with hemispherical cap and microballoon structure by using liquid core and TEOS as the template silica source, respectively [77], where the rigidity of the silica shell was adjusted by the size of liquid core. Notably, selective etching is also used to adjust the rigidity of silica nanoparticles to produce asymmetric structure. For instance, organosilica nanobowls were fabricated through a preferential etching approach (Fig. 7a) [78]. Because of the highly cross-linking of Si–OH groups on the surfaces, the interiors of mesoporous organosilica spheres were preferentially etched. After etching the cores completely and selectively etching the inorganic silicon in the shell, the frameworks were mainly dominated by the flexible Si–R–Si chains and non-cross-linked free Si-OX groups and were insufficient to support the original spherical structure, forming a bowl-like shape. Similarly, Zhang’s group also used this method to prepare mesoporous organosilica nanobowls [79]. More interesting silica nanoparticles with asymmetric structures also can be prepared via this etching strategy. The eccentric single-hole mesoporous nanocages have been designed by Zhao’s group. The SiO_2_@PMO composites were first prepared by anisotropically encapsulating the PMO on dense SiO_2_ nanoparticles. After removing the SiO_2_ nanoparticles, the eccentric hollow PMO nanoparticles were formed. Finally, a single-hole structured silica nanoparticles were formed after further etching by HF (Fig. 7b) [80].

The interfacial assembly, especially oil–water interface, is another effective strategy to prepare asymmetric silica nanoparticles. In 2011, Kuijk et al. prepared bullet-shaped asymmetric silica nanomaterial with tunable length by using an mixed solution of pentanol–water as solvent, and the growth process was recorded by transmission electron cryomicroscopy (cryo-TEM) at different reaction times [83]. It was found that the hydrolyzed TEOS only presented in the water-rich emulsion droplets, the nucleus grown in one direction after nucleation at the oil–water interface. And the bullet-shaped asymmetric silica nanoparticles were obtained due to the surface tension. Inspired by this work, Wang et al. used a similar method designed for the tadpole-like nanowires by introducing the surfactant trimethoxy(octadecyl)silane [84]. Later, bottle-like silica nanoparticles were also successfully prepared using two precursors, (3-chloropropyl)trimethoxysilane (CPTMS) and tetrabutylorthosilicate (TBOS) in the water-n-pentanol system (Fig. 7c) [81]. In this study, a silica film was first formed on the surface of the water droplets by the polymerization of –Si(–O^−^)_3_, which was produced by the hydrolysis of CPTMS. Then, the bowl-shaped silica shell was formed when TBOS was condensated on the silica film formed above. As the precursor continues to polymerize on the surface of the water droplet, the thickness of the shell increased and the water droplet was squeezed to form a new oil–water interface; then, the precursors continue to polymerize at the newly formed oil–water interface, obtaining a flask-like structure with an opening. Nonetheless, constructing asymmetric silica nanoparticles with a highly controllable dendritic silica compartment with large pores is still a great challenge. Inspired by the hydrolysis and condensation principle of TEOS in water/oil biphasic systems, Yu and co-workers successfully designed the badminton-like mesoporous silica (Fig. 7d) [82]. In a typical synthesis, monodispersed silicas as head particles were dispersed in a water–chlorobenzene system, TEOS as silica source, triethanolamine (TEA) as a catalyst, and cetyltrimethylammonium chloride (CTAC) was used as a structure-directing agent. Owing to the electrostatic attraction, the surface of head particles changed to positively charged after CTA^+^ modification. The negatively charged silicate species produced by hydrolysis and condensation of TEOS preferentially nucleated and grown on the surface of positively charged head particles near the water phase. Thus, the head–tail mesoporous silica nanoparticles were fabricated. The morphology, particle size, and tail length can be facilely tuned by varying the volume of TEOS, the amounts of head particles, and reaction time.

### Asymmetric Silica-Based Nanoparticles

In order to achieve more complex functionalities and extend their applications, different silica-based composite nanoparticles have also attracted widespread attention. Compared with conventional core–shell silica-based nanoparticles, a remarkable advantage of asymmetric silica nanostructure is that it can combine individual components in different spaces of single particle without interfering their optical, magnetic, and electronic properties. Therefore, different ingredients can work together to achieve multiple functions. According to the synthetic mechanism, the methods for silica-based asymmetric structures roughly include two strategies, namely interface modification and nucleation control.

**Fig. 8:**
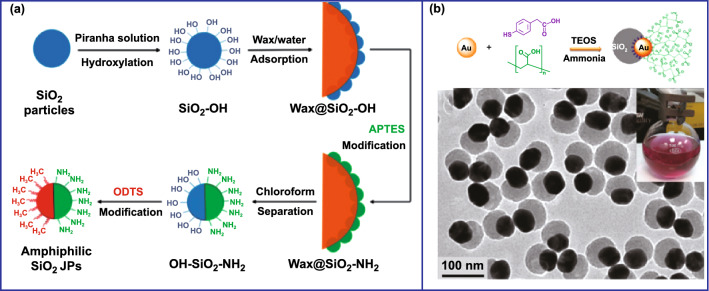
Preparation of various asymmetric silica-based nanoparticles. **a** Preparation of amphiphilic Janus silica particles by Pickering emulsion. Reproduced with permission from Ref. [100]. Copyright 2015, American Chemical Society. **b** Preparation of Janus Au-SiO_2_ nanoparticles by selective modification. Reproduced with permission from Ref. [106]. Copyright 2010, American Chemical Society

The construction of the Pickering emulsion interface has been regarded as one of the most basic methods to prepare asymmetric silica-based nanoparticles [85–98]. The paraffin/water interface is one of the most commonly used Pickering emulsion interfaces [99]. He et al. used the emulsion of paraffin and water to fix the hydroxylated silica particles, leaving half encapsulated in paraffin and another half exposed outside. Then, the exposed surface of silica was functionalized with amino groups. Finally, paraffin was removed to obtain Janus silica particles modified by amino and hydroxyl on both sides, respectively (Fig. 8a) [100]. In addition, other functional materials can be selectively grown on the silica surface following this method [101–104]. For example, gold–mesoporous silica Janus nanoparticles were successfully fabricated. The mesoporous silica nanoparticles were firstly fixed onto the emulsion interface of paraffin and water [105]. By partially functionalized with thiol groups by the addition of (3-mercaptopropyl)trimethoxysilane on the exposed surface, gold nanoparticles were selectively grown on it by the formation Au–S bonds to fabricate the Janus gold–mesoporous silica nanoparticles after removing paraffin.

Selective modification is also a direct way to endow the seeds with different surface properties for the growth of Janus structure [107–109]. For instance, Chen et al. simultaneously modified Au nanoparticles with 4-mercaptophenylacetic acid (4-MPAA) and poly(acrylic acid) (PAA_86_) [110]. Because of the different properties of the modifiers, the silica species preferentially grew onto the 4-MPAA modified side of gold seeds rather than the PAA-modified side, thereby obtaining Janus silica–Au nanoparticles (Fig. 8b). Kane et al. simultaneously modified the Au nanoparticles with PAA and 4-mercaptobenzoic acid (4-MBA) to render the surface of the gold seeds with different properties based on the similar strategy [106]. Owing to the high interfacial energy of the gold–ligand–solution interface, only the 4-MBA-modified part of Au nanoparticles can promote heterogeneous nucleation of silica to produce asymmetrically coated Au nanoparticles. The researchers also demonstrated that silica–gold nanoparticles with the core–shell structure can be achieved when only 4-MBA modification was performed, and further confirmed the critical role of selective modification for generating Janus structure.

**Fig. 9:**
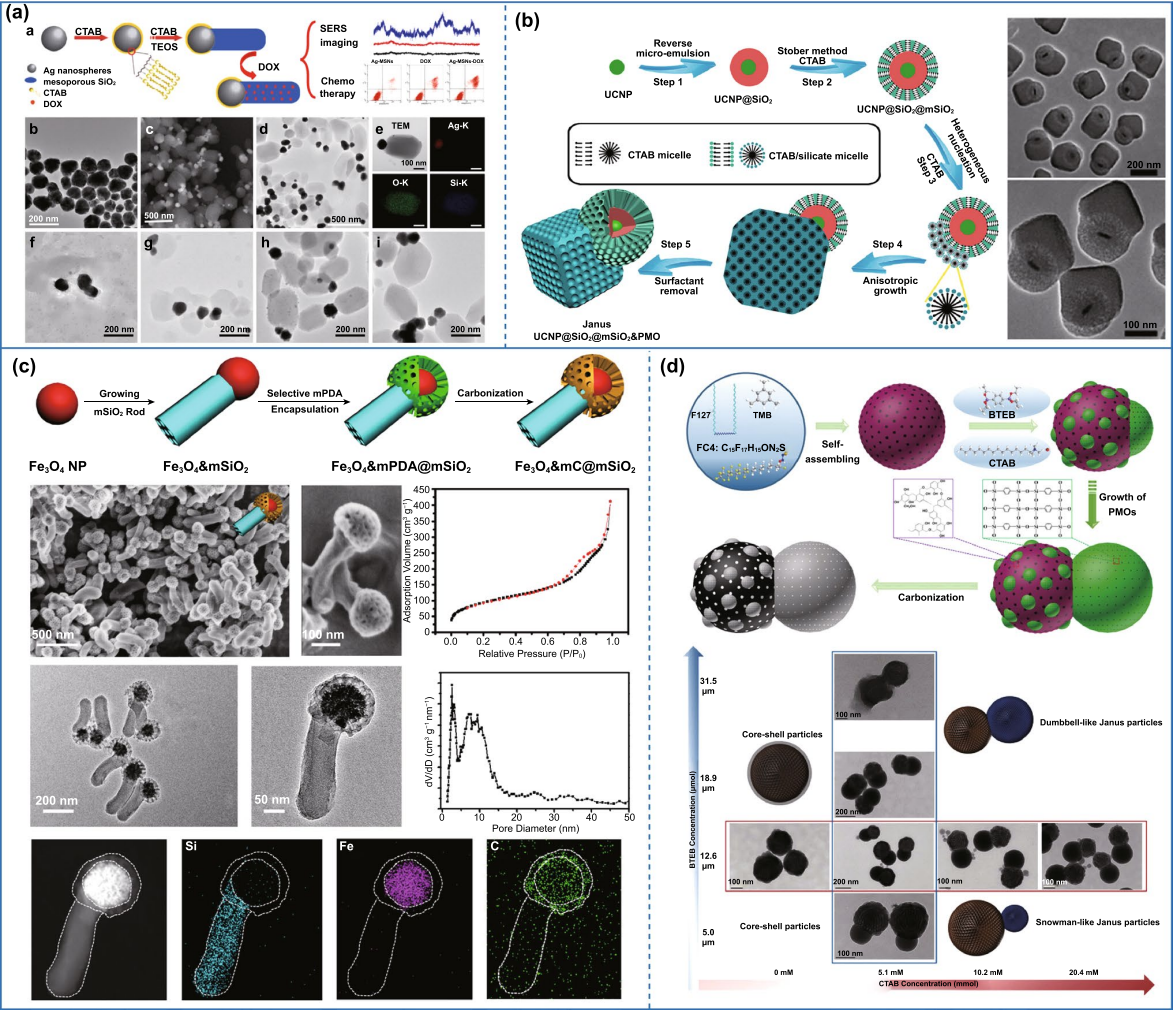
Preparation of various asymmetric silica-based nanoparticles. **a** Fabrication of Janus Ag-silica nanoparticles through nucleation control by the regulation of the reaction solvent. Reproduced with permission from Ref. [119]. Copyright 2016, American Chemical Society. **b** Preparation of the multifunctional dual-compartment Janus silica nanocomposites through nucleation control. Reproduced with permission from Ref. [122]. Copyright 2014, American Chemical Society. **c** Fabrication of Janus structure through interface modification and nucleation control. Reproduced with permission from Ref. [125]. Copyright 2018, American Chemical Society. **d** Preparation of Janus structure by regulating the concentration of surfactants. Reproduced with permission from Ref. [120]. Copyright 2017, Wiley-VCH

Apart from these, some unique asymmetric silica-based nanoparticles can be fabricated by surface-controlled nucleation and growth. Generally, the nucleation and growth method are determined by the overall Gibbs free energy, which can be represented as $\Delta \sigma = \sigma_{2 - s} - \sigma_{1 - s} + \sigma_{1 - 2}$, where σ_2‑s_ is the interfacial energy between component 2 and solvent, *σ*_1‑s_ is the interfacial energy between substrate and solvent, *σ*_1‑2_ is the interfacial energy between substrate and component 2. When Δ*σ* > 0, it is difficult for component 2 to diffuse on the surface of substrate, thus resulting in an asymmetric structure. The choice of the reaction solvent was an effective method to control the overall Gibbs energy for preparing the Janus structure. In 2011, Sun's group prepared the Fe_3_O_4_–SiO_2_ Janus particles with an accurately adjustable aspect ratio through the control of the reaction solvent for the first time [111]. In the synthesis, the traditional core–shell structure of Fe_3_O_4_@silica was synthesized when ethanol was added, further illustrating the importance of the reaction solvent. Unfortunately, the work did not clarify the growth mechanism of this unique Janus structure from a deep insight. Driven by this work, Ag–SiO_2_ [112, 113], Fe_3_O_4_–SiO_2_ [114], Au–SiO_2_ [115–117], and other [118] silica-based Janus composites have been synthesized. Later, Chen's group prepared Janus Ag–mesoporous silica nanoparticles and explained the formation mechanism using the theory of surface free energy (Fig. 9a) [119]. In this system, the total surface free energy can be represented as $\Delta \sigma = \sigma_{{{\text{msio}}_{2} - {\text{water}}}} - \sigma_{{{\text{Ag}} - {\text{water}}}} + \sigma_{{{\text{Ag}} - {\text{msio}}_{2} }}$, and the surface free energy of mSiO_2_ $\sigma_{{{\text{msio}}_{2} - {\text{water}}}}$ in the pure water system was significantly higher than that in alcohol–water system. Therefore, the total surface free energy Δ*σ* > 0, which would hinder the diffusion of CTAB/silicate micelles on Ag cores. After the nucleation site was formed, since the energy barrier of heterogeneous growth is much greater than that of homogeneous growth, mSiO_2_ will grow asymmetrically on the nucleation site instead of gradually covering the Ag core to form a symmetric structure. Finally, the core–shell structured Ag–mSiO_2_ Janus nanocomposites were realized. In addition to those simple silica-based asymmetric structures mentioned above, it has been a long-term goal to achieve more complex and multifunctional silica-based asymmetric structures by adjusting the interfacial tension. Surface-controlled nucleation and growth was the most effective strategy to fabricate complex asymmetric silica-based nanoparticles [47, 120, 121]. For instance, the multi-functional dual-compartment Janus silica nanocomposites UCNP@SiO_2_@mSiO_2_&PMO (UCNP, = NaGdF_4_:Yb, Tm@NaGdF_4_,) were prepared by anisotropic island nucleation and growth (Fig. 9b) [122]. In this case, the ratio of water to ethanol played an important role to the total surface energy of the system. Moreover, when the volume ratio of H_2_O: ethanol was reached to 15: 1, causing Δ*σ* > 0, it is difficult for CTAB/silicate micelles to diffuse on the surface of UCNP@SiO_2_@mSiO_2_, Thus, the asymmetric structured UCNP@SiO_2_@mSiO_2_&PMO nanoparticles were formed. Inversely, the concentric core@shell@shell@shell structured UCNP@SiO_2_@mSiO_2_@PMO nanocomposites were obtained when the volume ratio between H_2_O and ethanol was decreased to 2: 1 due to the Δ*σ* < 0. After that, other complex and multifunctional asymmetric silica-based nanoparticles had been designed by the same group [123, 124]. For example, dual-mesoporous Fe_3_O_4_@mC&mSiO_2_ Janus magnetic nanoparticles with tunable hydrophilic/hydrophobic ratio were designed, recently (Fig. 9c) [125]. First, the Fe_3_O_4_–SiO_2_ Janus nanoparticles were fabricated through the regulation of interface energy. Subsequently, mesoporous polydopamine was selectively modified on Fe_3_O_4_ segment through electrostatic interaction. Finally, dual-mesoporous Fe_3_O_4_@mC&mSiO_2_ Janus nanoparticles were fabricated by high-temperature carbonization treatment. More recently, the same group reported an interesting strategy called “surface-kinetic-mediated multi-site nucleation” for synthesizing asymmetric mesoporous multipods composed of a centering core@shell Fe_3_O_4_@SiO_2_@RF nanoparticle and four surrounding PMO nanocubes as pods [124]. The results demonstrated that the heterogeneous nucleation kinetics of oligomers on the substrate can be precisely regulated by changing the surface functional groups of RF layers. Consequently, the number of nucleation sites at the beginning of reaction significantly increased with the increase in the nucleation kinetics, thereby enabling the uniform growth of one pod, two plane-distributed pods, tripods, tetrapods, or even multi-pods on the centering substrate.

In addition to the solvent, the selection of surfactants also can regulate the total interfacial energy in reaction system. Liu's group used resorcinol–formaldehyde (RF) nanospheres as the seeds and 1,4-bis(triethoxysilyl)benzene (BTEB) as the silica source to prepare silica–RF nanocomposites (Fig. 9d) [120]. The structure of the nanocomposites can be changed by regulating the amount of surfactant CTAB. Specially, only core–shell structure was formed in the absence of CTAB, while a dumbbell-shaped Janus structure can be prepared with the increased amount of CTAB.

Some new synthetic techniques can also be used to prepare some emerging asymmetric materials [126–128]. For instance, zinc-doped indium oxide has been successfully printed on the silica surface to obtain asymmetric 2D silica nanocomposite [129].

## Applications of Asymmetric Structure of Carbon- and Silica-Based Nanoparticles

By virtue of the unique advantages of asymmetric structure, such as larger effective surface area, controllable structure and composition, the asymmetric carbon- and silica-based nanoparticles have attracted increased interest in various application fields in the past decade (Table 1). In this section, we will focus on the applications of asymmetric carbon- and silica-based nanoparticles in energy, catalysis, biomedicine, sensor, and other related directions, in particular highlighting the structural advantages from the viewpoint of structure–performance relationship.

**Table 1:** A summary of synthetic methods, morphology and structure, and their different applications of asymmetric carbon- and silica-based nanoparticles

Materials	Morphologies and structures	Synthetic methods	Applications	References
N-doped porous carbon	Bowl	Hard template	Li-S batteries	[11]
MoSe_2_/carbon	Pistachio-shuck	Self-template	K-ion batteries	[43]
Carbon	Nano-cups	Chemical vapor deposition	Li-ion batteries	[45]
Carbon	Bowl	Soft template	K-ion batteries	[130]
NiO@carbon	Bowl	Self-template	Li-ion batteries	[131]
SnO_2_@carbon	Bowl	Self-template	Li-ion batteries	[132]
Mesoporous α-Fe_2_O_3_/C	Bowl	Soft template	Li-ion batteries	[133]
CoS/C	Bowl	Self-template	Na-ion batteries	[134]
C@MoS_2_	Bowl	Hard template	Li-ion batteries	[135]
C@FePO_4_@rGO	Bowl	Hard template	Na-ion batteries	[136]
Nitrogen-doped carbon	Bowl	Soft template	Supercapacitors	[21]
Carbon	Bowl	Soft template	Supercapacitors	[22]
Carbon	Bowl	Self-template	Supercapacitors	[29]
Carbon	Bowl	Hard template	Supercapacitors	[32]
Carbon	Flask	Soft template	Supercapacitors	[40]
Porous carbon	Bowl	Hard template	Supercapacitors	[44]
N-doped porous C/NiCo_2_O_4_	Hemispherical	Soft template	Supercapacitors	[137]
Pt/C	Bowl	Hard template	Methanol oxidation and ORR	[28]
Co/N-doped carbon	Single-hole	Hard template	Oxygen reduction reaction	[36]
Porous carbon	Bowl	Soft template	Oxygen reduction reaction	[37]
Pt/Fe_3_O_4_@mC&mSiO_2_/NH_2_	Janus structure	Selective encapsulation strategy	Biphasic reduction of 4-nitroanisole	[125]
Pt/carbon/organosilica	Dumbbell	Wet chemical method	Two-phase nitroarene reduction reaction	[120]
Pd/C	Bowl	Hard template	Formic acid oxidation reaction	[138]
N/S co-doped Carbon	Bowl	Hard template	Oxygen reduction reaction	[139]
Fe_3_O_4_@C	Bowl	Soft template	Oxygen reduction reaction	[140]
Pt/NxC@mSiO_2_	Spherical	Hard template	Selective oxidation of alcohols	[141]
SiO_2_@PDVB/PS	Snowman	Seed emulsion polymerization	Reduction of 4-nitroanisole	[142]
Au/Fe_3_O_4_@C	Snowman	Surface-controlled nucleation	Targeted chemo-photothermal synergistic cancer therapy	[51]
UCNP@SiO_2_@mSiO_2_&PMO	Janus structure	Surface-controlled nucleation	Dual-drug delivery	[122]
Gold-mesoporous silica	Triangle bullet	Surface-controlled nucleation	Therapy of liver cancer	[115]
Fe_3_O_4_@mesoporous silica	Bullet	Surface-controlled nucleation	Targeting liver cancer chemotherapy	[143]
Ag/silica	Snowman	Surface-controlled nucleation	Liver cancer chemo/photothermal therapy	[144]
Au-mesoporous silica	Snowman	Surface-controlled nucleation	Chemo-photothermal treatment of liver cancer cells	[145]
Silica	Bowl	Hard template	DNA delivery	[146]
Fe_3_O_4_-silica	Bullet	Surface-controlled nucleation	Anti-metastatic immunotherapy	[147]
Au-silica	Spherical	Selective modification	Self-propelled motors	[148]
Gold-mesoporous silica	Snowman	Surface-controlled nucleation	Logic gate	[149]
Gold-mesoporous silica	Snowman	Surface-controlled nucleation	Interactive models of communication	[150]
γ-Fe_2_O_3_ and silica-GOx	Hamburger	Flame-assisted spray pyrolysis	Sensors for colorimetric detection of glucose	[151]

### Electrochemical Energy Storage Applications

In the contemporary environment of pursuing low-carbon life, the use of new renewable clean energy instead of the fossil fuels has emerged. The electrochemical energy storage devices have been widely deemed as one of the best types of energy storage in various new energy sources due to its good stability, recyclability, and no memory effect. According to the energy storage mechanism, the electrochemical energy storage device is mainly divided into reversible secondary batteries (e.g., known lithium-ion batteries, LIBs) and supercapacitors [152–158]. As one of the main components of energy storage device, it has been demonstrated that the structure and composition of electrode materials including cathode and anode seriously affect their electrochemical performances.

Carbon- and silica-based nanoparticles, especially carbon nanoparticles, have become the main electrode materials due to the good conductivity, large surface area, and excellent electrochemical stability [159–165]. Also, the rational design of materials structure can effectively improve its energy storage performance [166–168]. The traditional hollow structure is one of the most ideal structures because of its tunable inner cavity, which can effectively alleviate the large-volume expansions during charge–discharge processes. However, it also suffers from a limited volumetric energy density owing to a larger internal cavity, resulting in a smaller bulk density. Additionally, a larger internal cavity will reduce the structural stability of the electrode materials, thereby leading to poor cycling stability and low capability. In contrast, the asymmetric structure can effectively alleviate the above shortcomings and exhibit some unique new functions benefiting from its unique structural merits.

**Fig. 10:**
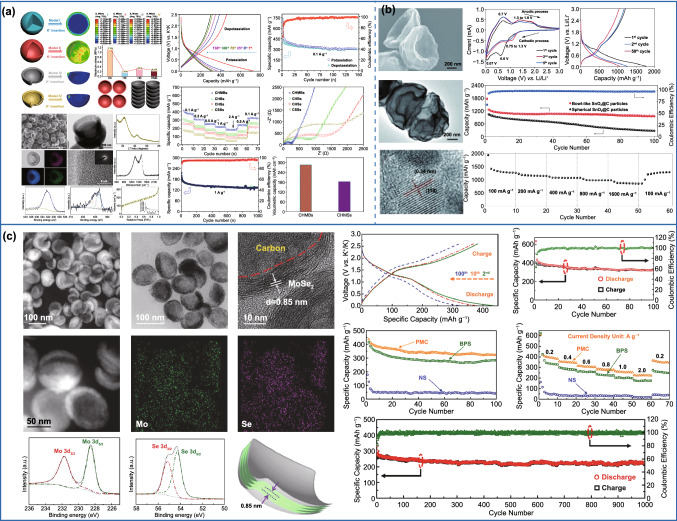
Application of asymmetric structure in secondary batteries. **a** Application of asymmetric pure carbon in secondary batteries. Reproduced with permission from Ref. [130]. Copyright 2019, American Chemical Society. **b** Application of asymmetric SnO_2_@C composite material in Li-ion batteries. Reproduced with permission from Ref. [132]. Copyright 2014, Wiley–VCH. **c** Application of asymmetric MoSe_2_/C composite material in K ion batteries. Reproduced with permission from Ref. [43]. Copyright 2019, American Chemical Society

For the electrode materials, the larger effective specific surface area provided by asymmetric structures can maximize the electrolyte permeation and enhance the contact area between electrode and electrolyte interfaces, which can effectively boost the electrochemical performances. For example, Zheng et al. designed porous bowl-like N-doped carbon with an extremely high BET surface area of 2161 m^2^ g^−1^ [11]. When as cathodes for Li–S batteries, the obtained S/N-HPCB electrode showed a high reversible capacity of 894 mAh g^−1^ at 0.1 C, and it still retained 706 mAh g^−1^ after 400 cycles at 1.0 C. The superior electrochemical performance was attributed to efficiently immobilize sulfur and polysulfide provided by the high surface area of bowl-like structure, leading to maximum interaction between sulfur and carbon host and minimal dissolution of lithium polysulfide intermediates. Other asymmetric carbon nanoparticles have also been designed as electrode materials for reversible secondary batteries [32, 45, 46]. For instance, Jian et al. prepared carbon nanocups through a facile catalytic chemical vapor deposition (CCVD) technique [45]. Benefiting from the enhanced spacings for ionic storage and mass transport of unique opening structure, the resulting carbon cups delivered a high reversible capacity of 953 mAh g^−1^ after 100 cycles at 50 mA g^−1^ and 468 mAh g^−1^ after 100 cycles at 25 mA g^−1^, respectively, for LIBs and sodium-ion batteries (SIBs). Compared with LIBs and SIBs, more severe volumetric changes on electrode materials during the intercalation of K ions have become the main obstacle for obtaining the stable electrode materials capable of undergoing long-term potassiation/depotassiation, caused by the larger radius of K ions (1.38 Å). To address the issue, bowl-like asymmetric hollow multi-hole carbon nanoparticles (CHMBs) were prepared and systematically explored the stress response by simulating the von Mises stress distributions (Fig. 10a) [130]. The result showed that the maximum stress of hollow multi-hole bowl was 0.254 MPa, considerably smaller than that of the solid sphere (1.154 MPa) and a little higher than that of the hollow sphere (0.184 MPa). However, CHMBs had a higher tap density in comparison with the hollow sphere. Consistent with the above simulation results, the CHMBs electrode exhibits an excellent durability and a high reversible capacity of 304 mAh g^−1^ at 0.1 A g^−1^ due to the shortened electron/ion transport distance. Especially, the volumetric specific capacity of CHMBs was 56% higher than that of hollow carbon spheres due to the higher pack density. Similar to other symmetrical nanoparticles, the electrochemical performance of asymmetric carbon nanoparticles can also be further improved by introducing heteroatoms or compounding with other high-capacity materials [169–175]. For example, bowl-like carbon hybridized with SnO_2_ nanosheets (SnO_2_@C) has been designed as electrode materials for lithium storage (Fig. 10b) [132]. Thanks to the unique structure and composition advantages, the as-made SnO_2_@C electrode demonstrated a high reversible capacity of 963 mAh g^−1^ at 0.4 A g^−1^ after 100 cycles. Analogously, various asymmetric carbon-based composite nanoparticles, including C-NiO [131, 176], C-Fe_3_O_4_ [133], C-CoS [134], C-MoS_2_ [135, 177–179] and C-FePO_4_-RGO [136], have been designed as electrode materials for reversible secondary batteries. Nevertheless, most of them were compounded in two steps, and the synthesis process is relatively complicated and time-consuming. Recently, Guo’s group prepared pistachio-shuck-like asymmetric MoSe_2_/C composite (PMC) based on a simple one-step process (Fig. 10c) [43]. The unique structure can not only improve the diffusion of K-ions and increase the electrons and ions transfer, but also effectively enhance the volumetric energy density. The resulted PMC exhibited a high capacity of 322 mAh g^−1^ at 0.2 A g^−1^ over 100 cycles and excellent cycle stability.

**Fig. 11:**
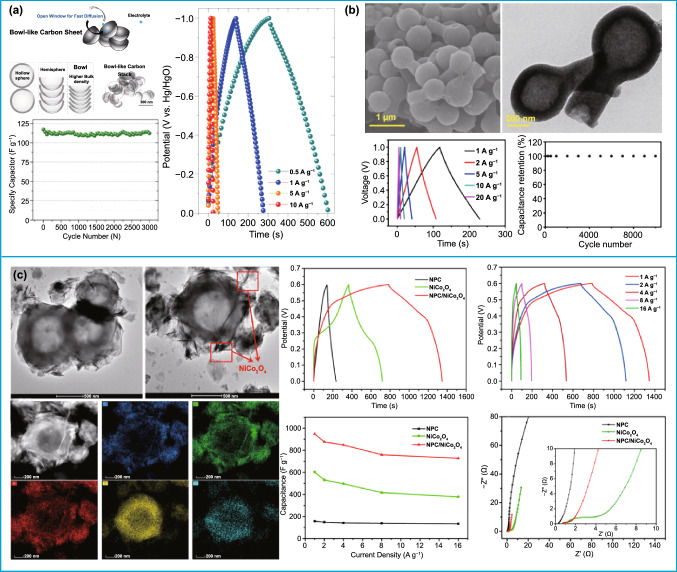
Application of asymmetric structure in capacitors. **a** Application of asymmetric pure carbon in capacitor. Reproduced with permission from Ref.[29]. Copyright 2014, Elsevier B.V. **b** Application of flask-like pure carbon in capacitor. Reproduced with permission from Ref. [40]. Copyright 2017, American Chemical Society. **c** Application of asymmetric hemispherical N-doped porous carbon/NiCo_2_O_4_ composite material in capacitor. Reproduced with permission from Ref. [137]. Copyright 2019, Elsevier B.V

Supercapacitors are another common electrochemical energy storage device. However, achieving high power and large energy capacity at a high rate is still difficult in the development of electrochemical capacitors owing to the primary kinetic limits of electrochemical processes in electrode materials. Benefiting from the large surface area, higher bulk density, and better structural stability, asymmetric carbon-based nanoparticles demonstrated a fascinating application prospect in supercapacitors. For instance, bowl-shaped carbon nanosheets have been used as an electrode material for supercapacitors (Fig. 11a) [29]. In virtue of the merits including higher bulk density, interconnected channel, and hierarchical porosity of thin-walled bowl-like sheet, the prepared carbon sheet had a high specific capacitance of 151 F g^−1^ at 0.5 A g^−1^. Similarly, a series of asymmetric bowl-like carbon nanoparticles have also been designed as electrode materials with excellent electrochemical performance for supercapacitors [21, 22, 32, 34, 44]. Besides, a unique flask-like hollow carbonaceous electrode material was also synthesized, exhibiting a high specific capacitance of 263 F g^−1^ at 0.1 A g^−1^ and 222 F g^−1^ at 1.0 A g^−1^. Importantly, the obtained carbon electrode material had no decrease in capacitance after 10,000 cycles at 20 A g^−1^. The superior electrochemical performance can be attributed to the larger surface area (2335 m^2^ g^−1^), pore volume (1.34 cm^3^ g^−1^), and better structural stability provided by the unusual hollow and open structure, which can effectively improve the charge storage and accelerate ion transport (Fig. 11b) [40]. However, pure carbon nanoparticles still exhibited a low theoretical capacity. Similar to the secondary batteries, it was also one of the most effective ways to improve the energy storage capacity of supercapacitors by coupling with other high-capacity materials [180–183]. Recently, Ma’s group got a hemispherical N-doped porous carbon/NiCo_2_O_4_ (NPC/NiCo_2_O_4_) composite (Fig. 11c) [137], where the hemispherical N-doped porous carbon as buttress to restrain NiCo_2_O_4_ nanosheets from aggregation and NiCo_2_O_4_ as covering layer to enhance the capacitance of carbon. When as the electrode material for supercapacitors, NPC/NiCo_2_O_4_ reached specific capacitance of 948.30 F g^−1^ at 1.0 A g^−1^, and the capacitance retention of 87.4% at 10 A g^−1^ after 2000 cycles, manifesting an excellent durability.

### Catalytic Applications

**Fig. 12:**
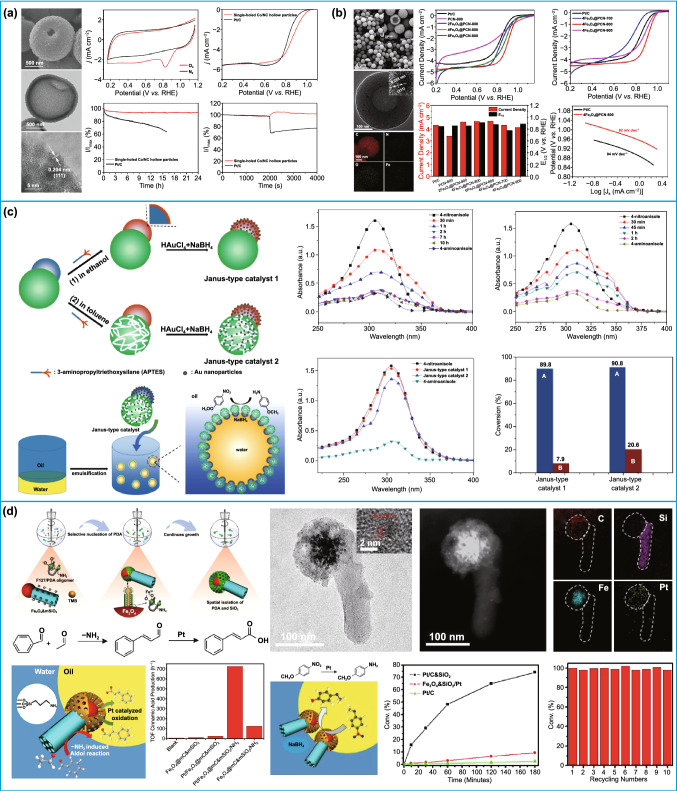
Application of asymmetric structure in catalysis. **a** Single-hole cobalt-/N-doped carbon hollow particles for oxygen reduction reaction. Reproduced with permission from Ref. [36]. Copyright 2017, Wiley–VCH. **b** Fe-embedded porous nanobowls for ORR. Reproduced with permission from Ref. [140]. Copyright 2019, Elsevier B.V. **c** Application of the SiO_2_@PDVB/PS Janus particles in two-phase catalytic reaction. Reproduced with permission from Ref. [142]. Copyright 2017, Elsevier B.V. **d** Application of the spatial isolation silica–carbon Janus structure complex in two-phase catalytic reaction. Reproduced with permission from Ref. [125]. Copyright 2018, American Chemical Society

As we known, the catalytic reaction generally takes place at the interface between reactants and catalyst. This means that only the active ingredients that are exposed to reactants are highly active. Thus, the performance of the catalyst material is closely related to their surface morphology and internal structure [184–189]. Traditional hollow structures have showed a good application prospect in the catalytic fields in virtue of their large surface area and low density [190–194]. Nonetheless, the larger internal space usually results in a lower effective surface area and a smaller bulk density. In contrast, the unique advantages of asymmetric structures, e.g., larger effective surface area, can significantly provide more active sites for substrates, thereby boosting their catalytic performance [19, 28, 138]. For example, bowl-shaped carbon nanoparticles (BLCs) with large surface area (1108.3 m^2^ g^−1^) and large pore volume (2.7 cm^3^ g^−1^) have been applied as supporting materials of noble Pt for oxygen reduction reaction (ORR) in acidic media [28]. Owing to the synergistic effect between the uniform bowl-like structure and uniform and stable loading of smaller Pt particles, the Pt/BLC electrocatalyst exhibits much higher electrocatalytic activity and stability. The mass current densities were 1.6 times for ORR as high as that of commercial Pt/C. In addition, the catalytic performance can be further improved by controlling the chemical compositions of catalysts [195–198]. Lou's group designed single-hole cobalt-/*N*-doped carbon hollow particles for oxygen reduction reaction (Fig. 12a) [36], which exhibited a superior electrocatalytic performance. Similarly, N or N and S co-doped bowl-like carbon nanoparticles also have been designed as electrocatalysts for oxygen reduction reaction [37, 139]. However, the above catalysts have a lower half-wave potential compared with commercial Pt/C catalysts. Recently, Fe-embedded porous nanobowls were prepared for ORR (Fig. 12b) [140]. The obtained Fe_3_O_4_@PCN-800 catalyst had the higher half-wave potential (0.911 V vs. RHE) than Pt/C (0.845 V vs. RHE). On the other hand, the asymmetric structures also exhibit a higher electrocatalytic activity for the methanol electro-oxidation. Hollow carbon hemispheres were prepared as the support material for designing Pt nanoparticle catalysts (Pt/HCHS) [19]. Thanks to the unique asymmetric structure, which were more favorable for the dispersion of Pt nanoparticles, the obtained Pt/HCHS catalysts showed a superiority electrocatalytic activity for the methanol electro-oxidation in terms of the onset potential, current density, and stability in alkaline solution. Besides, benefiting from the unique structure advantages, the asymmetric structure as supporting materials can significantly improve the reactivity of single-metal electrocatalyst for the formic acid oxidation reaction. The high-quality carbon nanobowls (CNBs) with high surface area were designed as advanced supporting material to anchor Pd nanocrystals for formic acid oxidation reaction [138]. The prepared Pd/CNBs nanohybrids exhibited much higher formic acid oxidation reaction activity and durability than commercial Pd/C electrocatalyst due to the uniform dispersion of the Pd nanocrystals, which were beneficial from the high surface area and unique structure advantages of bowl-like structure.

In addition to the above electrocatalysis, biphasic catalysis is another important catalytic reaction [199–203]. The reactions are carried out in a biphasic mixture of two immiscible solvents for biphasic catalysis. As a result, the emulsion stability, efficient reaction interface, and recycling of catalysts are the major objectives of biphasic catalysis [86, 204–211]. The asymmetric structure exhibits irreplaceable application potential in biphasic catalytic because of tunable amphiphilicity, which can not only stabilize the emulsion and increase catalytic efficiency, but also effectively simplify the reaction process [212–214]. In 2010, Crossley's group reported the application of Janus-structured recoverable catalyst in two-phase interfacial catalysis for the first time [215]. In this work, the emulsions’ stabilization and biphasic hydrodeoxygenation and condensation can be simultaneously achieved by selectively depositing Pd onto either the hydrophilic silica oxide or the hydrophobic nanotubes of the carbon nanotube–silica. Since then, Janus nanoparticles with different structures were well developed as catalysts for two-phase interfacial catalysis [141]. For instance, Liu's group prepared snowman-like SiO_2_@PDVB/PS Janus catalysts with Au nanoparticles selectively modified on one or both heads to systematically investigate the catalytic performance through the reduction of 4-nitroanisole (Fig. 12c) [142]. Owing to the larger reaction area and good emulsification property, the as-prepared Janus-type catalysts not only exhibited excellent catalytic activity in homogeneous reaction system, but also more efficient catalytic activity at water-in-oil emulsion interface. Among these asymmetric morphologies, the dumbbell-shaped Janus particles exhibit some unique advantages in exquisite controlling the chemical reaction of the two-phase mixture because they tend to assemble in a single direction perpendicular to the oil–water interface [216]. Pt-loaded dumbbell-shaped mesoporous carbon–PMO Janus nanoparticles were prepared by the regulation of interface energy and used as catalysts for nitroarene reduction in water and toluene mixture solvents [120]. The nitrobenzene was fully converted to aniline under the stir-free conditions after 6 h. The superior catalytic efficiency can be ascribed to the short diffusion distances, controlled reaction location, and large reaction interfacial area provided by the unique dumbbell-shaped structure. Recently, dual-mesoporous Fe_3_O_4_@mC&mSiO_2_ Janus magnetic solid catalysts with absolute spatial isolation of carbon and silica were designed by Zhao's group (Fig. 12d) [125]. The resulting Janus nanoparticles showed outstanding performances in biphasic reduction of 4-nitroanisole with 100% conversion efficiency after electively anchoring catalytic active sites into different domains. Importantly, the catalysts can be easily recycled due to the magnetic functionality.

### Biomedical Applications

**Fig. 13:**
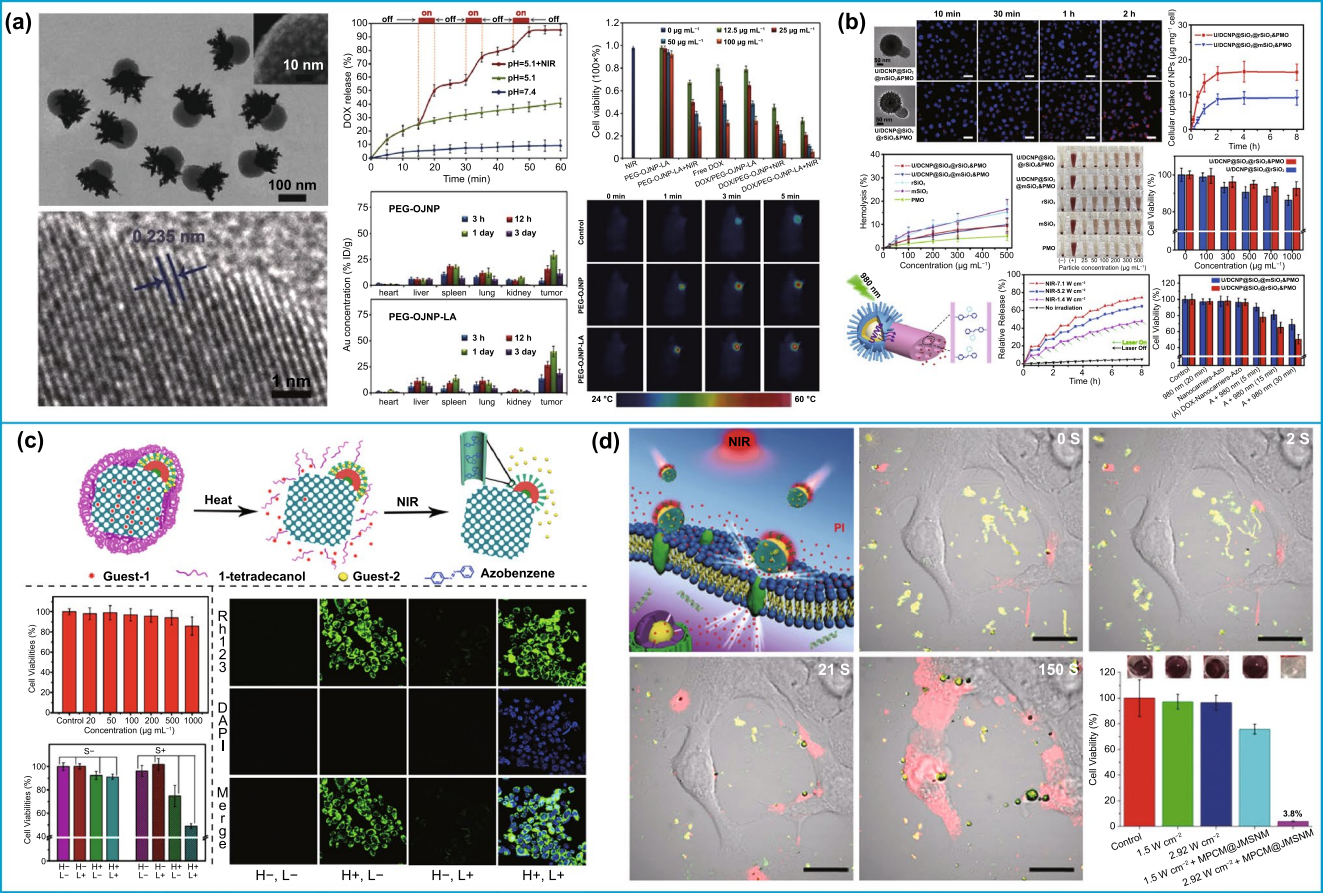
Application of asymmetric structure in biomedicine. **a** PEG-Au-PAA/mSiO_2_-LA Janus NPs for synergistic actively targeted and chemo-photothermal therapy. Reproduced with permission from Ref. [47]. Copyright 2016, Wiley-VCH. **b** Engine-trailer-structured nanotrucks for enhanced drug delivery. Reproduced with permission from Ref. [217]. Copyright 2020, Elsevier B.V. **c** Dual-compartment asymmetric silica nanocomposites for dual-drug delivery. Reproduced with permission from Ref. [122]. Copyright 2014, American Chemical Society. **d** MSNs-based nanomotor for thermomechanically percolating cell. Reproduced with permission from Ref. [148]. Copyright 2018, Wiley–VCH

With regard to biomedicine, the advantages of asymmetric nanoparticles lie in their possibility of integrating different functional components, structures, and even properties. The remarkable achievements of symmetric carbon- and silica-based nanostructures have greatly impelled the researchers to explore the biomedical applications of their asymmetric counterpart. Chen et al. prepared a bullet-like nanoparticle with a head of Fe_3_O_4_ and a body of mesoporous silica for drug delivery [143]. The magnetic Fe_3_O_4_ enables the magnetic field-guided tumor accumulation and enhanced the cellular uptake of Janus nanostructure, while mesoporous silica allows the efficient loading of antitumor drugs. Photosensitizers can also be loaded into the Janus Fe_3_O_4_–mesoporous silica, and Fe_3_O_4_ was utilized for combining magnetic hyperthermia with photodynamic therapy to potentiate the anti-metastatic immunotherapy [147]. By replacing the Fe_3_O_4_ segment with gold or silver nanoparticles, the resultant noble metal–silica asymmetric nanostructures are endowed with photothermal or antibacterial effect for synergistic photothermal/chemotherapy or antibacterial applications, respectively [113, 144, 145]. Moreover, the excellent radiosensitization and computerized tomography (CT) imaging capacity of gold are successfully imparted into the asymmetric nanostructure for multifunctional theranostics of tumor [115, 116]. To improve the delivery efficiency, lactobionic acid (LA) was also selectively conjugated onto the silica segments of octopus-type gold nanostar-mesoporous silica asymmetric nanoparticles. The obtained products not only possess high drug loading content, but also exhibit pH and near-infrared (NIR) dual-responsive release properties, which was used for actively targeted chemo-photothermal therapy (Fig. 13a) [47]. In comparison with silica-based nanoparticles, the biomedical applications of asymmetric carbon-based nanoparticles have rarely been reported. Wang’s group prepared a snowman-shaped nanostructure with ternary functional components of gold, Fe_3_O_4_, and carbon by controlling interfacial energy and selective modification [51]. Therefore, the multi-modality of CT/magnetic resonance (MR) imaging and chemo-photothermal synergistic therapy can be implemented in a single asymmetric nanoplatform.

These asymmetric nanoparticles can also exert its advantages in cargo delivery. Traditional mesoporous silica nanoparticles always show inferior loading efficiency for large-sized biomolecules like proteins and nucleic acids. To solve this problem, Qiao et al. developed bowl-shaped silica nanoparticles by nucleation control [146], which exhibited high loading capacity for plasmid DNA and great potential for DNA delivery applications. The cellular uptake of nanocarriers is also essential for efficient drug delivery. In a recent work, Li et al. proposed a asymmetric nanotruck with rough silica as “engine” and periodic mesoporous organosilica (PMO) rod as “trailer” for enhanced drug delivery (Fig. 13b) [217]. The silica head with rough surface significantly improves the intracellular uptake of the nanotruck compared with mesoporous silica nanoparticles with smooth surface. To realize precise therapy, upconverting nanoparticles (UCNPs) were also encapsulated into rough silica head for bio-imaging and on-demand drug release triggered by the NIR light. This ingenious design provides an inspiring paradigm of integrating the structural advantages in independent domains of asymmetric silica-based nanostructure.

In addition, the properties of asymmetric nanoparticles can be tailored to meet some specific requirements in biomedicine. Zhao et al. synthesized dual-compartment asymmetric silica nanocomposites with two segments of mesoporous silica and PMO for dual-drug delivery (Fig. 13c) [122]. More specifically, the hydrophilic property of mesoporous silica domains and hydrophobic property of PMO can accommodate two distinctly different spaces for separately loading hydrophilic and hydrophobic drugs without interfering each other. As a consequence, the bi-modal of heat and NIR light-triggered independent release of dual drug remarkably improves the cancer cell killing effect in comparison with single-triggered delivery system. Apart from the hydrophilicity/hydrophobicity, the surface of asymmetric nanostructure can provide different districts for specific functionalization. As an example, Vallet-Regí et al. reported the asymmetrically modification of MSNs with two targeted ligands, in which one side of folic acid binds with the receptors on cell membrane and another side of triphenylphosphine aim to the mitochondria [96]. This type of asymmetric modification ensures more specific and sequential targeting process of nanocarriers from cell to organelle, showing noteworthy advantages over traditional symmetric nanocarrier with homogeneous surface.

Another potential application of asymmetric nanoparticles was the exploration of nanomotors for active drug delivery. The asymmetric structure or selective modification enabled the self-propelled motion of nanomotors to fulfil the delivery task under complex biological conditions. MSN-based nanomotor was designed by selectively coated gold nanoshell onto the one side of silica surface (Fig. 13d) [148]. Upon NIR light irradiation, the half gold nanoshell converts the light into heat energy, generating a heat gradient on the Janus gold-mesoporous silica nanoparticle. Accordingly, the self-thermophoretic forces induced by anisotropic heating efficiently accelerate the internalization of Janus nanoparticles. Moreover, nanomotors powered by catalytic reaction have also been fabricated through selective deposition or modification of Pt or enzymes on MSNs [81, 218, 219]. Several studies claim that the autonomous motion of asymmetric silica-based motors can significantly improve their performance on penetrating cell membrane or even tumor tissue [105, 220, 221]. Based on these preliminary achievements, asymmetric nanoparticles are expected to fully exert their structural advantages in the emerging field of nanomotor, which urgently requires the development of new type of asymmetric nanoparticles with diversified composition, morphology, and functionality.

### Other Applications

**Fig. 14:**
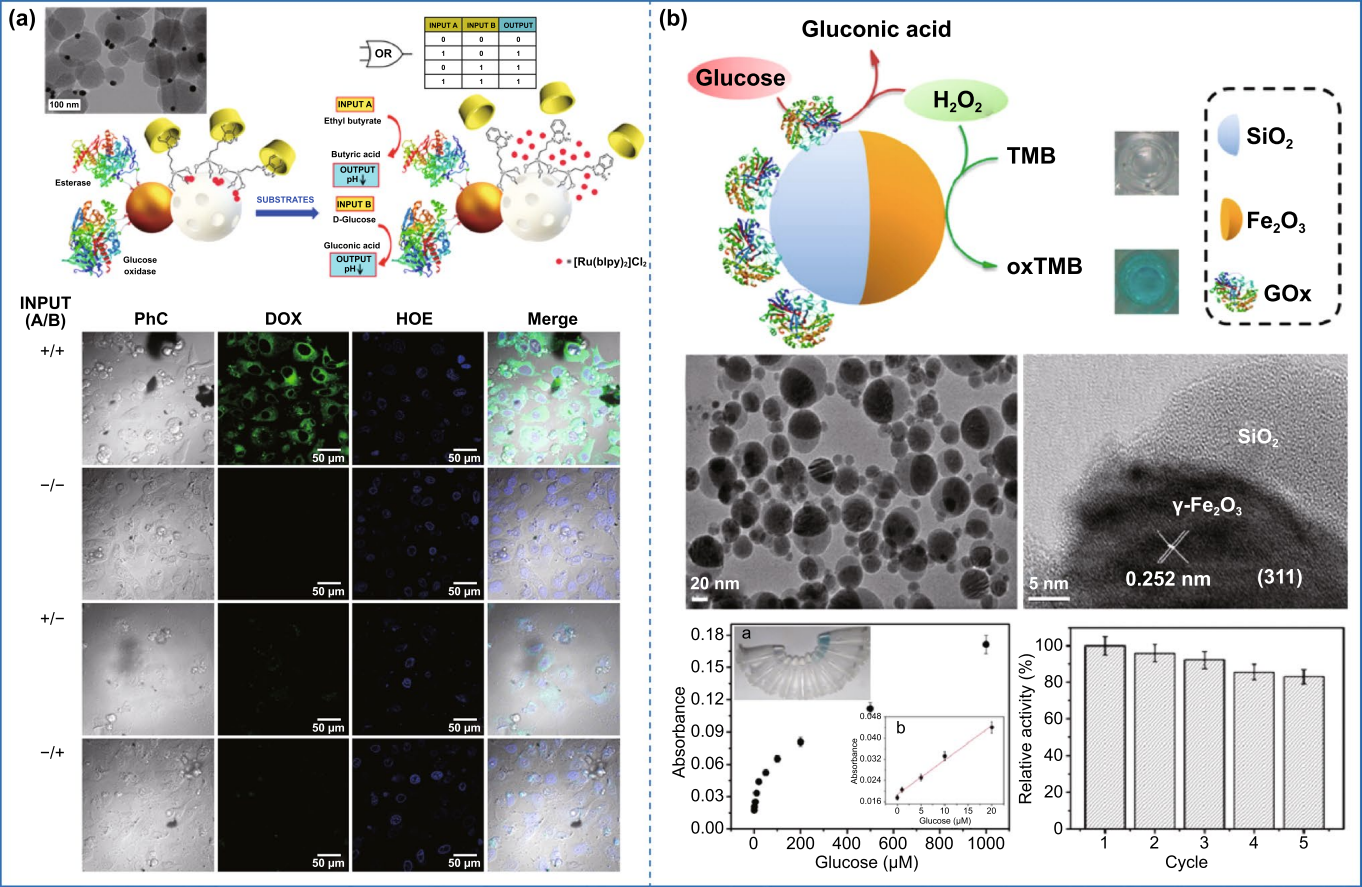
Application of asymmetric structure in others. **a** Application of Janus gold-mesoporous silica nanoparticles in logic gate. Reproduced with permission from Ref. [149]. Copyright 2014, American Chemical Society. **b** Application of Janus particles in sensors. Reproduced with permission from Ref. [151]. Copyright 2015, American Chemical Society

Apart from the above-mentioned directions, asymmetric nanostructures have also shown great potential for other interesting applications, like logic gate and sensors. Logic gates, presenting specific output signals in response to input stimuli, have drawn intensive interest in the area of molecular-scale electronics, and chemical and biological computers [222]. Generally, the fabrication of complex logic gates involves the integration of several gating systems and effectors in single nanoplatform. Asymmetric nanoparticles can perfectly achieve this goal, considering that Janus structures provide two different surfaces and enable conflicting functionalization process. Following this general concept, Pingarrón et al. prepared Janus gold-mesoporous silica nanoparticles as a “logic gate” controlled drug delivery system (Fig. 14a) [149]. The mesoporous silica segment can be served as a nanocarrier for cargo loading, and β-cyclodextrin (β-CD) was conjugated onto it as an acid-cleavable gatekeeper by supramolecular chemistry. On the side of the Janus structure, glucose oxidase (GOx) and esterase were attached onto gold surface as two effectors of “logic gate.” Therefore, the GOx and esterase can generate acid microenvironment under the catalytic decomposition reaction of glucose and ester, respectively, which subsequently induced the dissolution of supramolecular gatekeeper and open the mesochannel for drug release. This design utilizes an “OR” logic gate to govern the drug release in the presence of either stimuli in the system. In comparison, the “AND” logic gate can ensure more precise drug release behavior, which only occurs when two inputs are both delivered. However, the application of Janus structure for an “AND” logic gate-based system has not yet been developed. Beyond the logic gate, Martínez-Máñez and co-workers used the Janus gold-mesoporous silica to establish an interesting communication system based on the chemical reactions occurred on different compartments of the Janus structure [150]. Two nanomachines were prepared for this interactive model of communication: for the first nanomachine (S1_Gal_), the mesoporous silica was loaded with (Ru(bpy)_3_)^2+^ and modified with β-CD via disulfide bonds, and the gold side was immobilized with β-galactosidase; for the second nanomachine (S2_GOx_), the mesoporous silica segment was loaded with N-acetyl-L-cysteine and covered with β-CD through acid-sensitive bond, while the gold was immobilized with GOx. When the above two nanomachines encounter each other in solution, the communication process can be initiated by the addition of lactose. The β-galactosidase on S1_Gal_ catalyzes the hydrolysis of lactose into galactose and glucose. Subsequently, the resultant glucose diffused in the solution can be further decomposed to gluconic acid under the catalytic effect of GOx on S2_GOx_. The produced gluconic acid then makes the decrease in pH of the solution, causing the disassembly of supramolecular valves between *β*-CD and benzimidazole and further resulting in the diffusion out of *N*-acetyl-l-cysteine from the pores of mesoporous silica. Afterward, the released *N*-acetyl- l-cysteine acted as a feedback signal to induce the cleavage of the disulfide bonds on mesoporous silica segment of S1_Gal_. Finally, the uncapping of *β*-CD on S1_Gal_ induced the release of loaded (Ru(bpy)_3_)^2+^ into the system, which was considered as the only “output” signal for the communication of two nanomachines. This work forcefully demonstrated the advantages of asymmetric silica nanoparticles on diverse functionalization. Nevertheless, those communications were all based on the chemical reactions in solution system. Many other stimuli, including light, heat, magnetic field or even ultrasound, can also be used to construct remotely controlled “logic gate” or communication system for more intelligent applications, which encouraged the development of novel asymmetric silica-based nanoparticles combined with different functional materials.

It should be mentioned that symmetric nanoparticles often encounter difficulties on surface functionalization for sensing applications. For instance, nanoparticle-based sensors for capturing some biomarkers from cancer cells always required the conjugation of efficient targeting molecules to endow them highly specific binding capacity toward the certain cell lines. Additionally, attaching ligand or biomacromolecules onto the surface of nanoparticles would impair the detecting efficiency of the sensors to some degree. As mentioned above, asymmetric nanoparticles perfectly solve this issue by offering different districts for conflicting modifications [223]. For instance, Lu et al. recently developed a Janus-structured sensors for colorimetric detection of glucose (Fig. 14b) [151]. The Janus nanoparticles comprised two sides of *γ*-Fe_2_O_3_ and silica. The GOx was selectively modified onto silica surface by covalent bonds, while the *γ*-Fe_2_O_3_ segment acted as a catalyst for the chromogenic reaction. The detection mainly involves two steps: the GOx on silica side catalyzed the decomposition of glucose into gluconic acid and H_2_O_2_, and the Fenton reaction between generated H_2_O_2_ with the *γ*-Fe_2_O_3_ to generate products of distinct colors. The Fenton reaction can produce hydroxyl radical and further oxidize the tetramethylbenzidine (TMB) into a blue product. Therefore, the developed asymmetric *γ*-Fe_2_O_3_–SiO_2_ nanoparticles can not only accomplish the analytic mission, but also enable easy separation and recycling of the nanoparticles due to the magnetic response of Fe_2_O_3_ hemisphere.

## Summary and Outlook

**Fig. 15:**
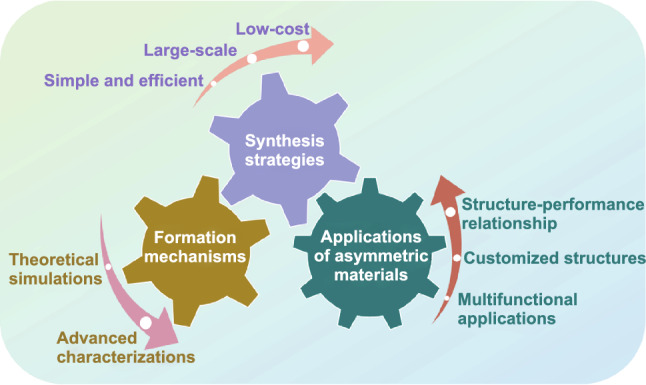
The main challenges and the future development prospects of asymmetric carbon- and silica-based nanomaterials

In recent years, asymmetric carbon- and silica-based nanomaterials have aroused more and more attention due to their attractive structure and composition advantages. The asymmetric structures with ingenious adjustability not only exhibit larger effective surface area accompanied with more active sites, but also are an ideal choice for designing “nano-intelligent systems” based on a single asymmetric particle. After decades of efforts, some preliminary results have been achieved in the design of asymmetric structures. Here, we mainly review the recent advances about the basic design principles and synthesis methods of asymmetric carbon- and silica-based nanomaterials. Meanwhile, the applications of different asymmetric structures in energy storage, catalysis, and biomedicine are discussed, and the performance advantages brought by their structures are also presented. Although some significant progress has been made in the design and application of asymmetric carbon- and silica-based nanomaterials, their development is still in the initial stage, and many challenges still need to be overcome (Fig. 15).

The first one is about the exploration of simple, efficient and universal synthesis strategies. Although some methods have been used to prepare different asymmetric structures, the current preparation techniques are relatively conventional and still have some inherent disadvantages. For instance, although the soft-templating route is relatively simple in experimental process, the resulting particles are usually large in size, often in the order of microns, while the hard-template method generally contains the tedious preparation process, resulting in high costs. Additionally, the current asymmetric structures, especially the carbon-based nanoparticles, are mainly limited to the simple bowl-shaped structure. How to easily and efficiently prepare the asymmetric structures with more complex morphology and functionalities is highly desirable. Furthermore, in most common methods for preparing asymmetric structures, only a small number of products can be got at a time. Meanwhile, the cost is another major problem need to be considered in the large-scale production. Thus, it remains a huge challenge for the preparation of asymmetric structures with uniform morphology and high yields. As a result, it is very meaningful to develop some new, facile, high-efficient, low-cost, large-scale routes to fabricate the high-performance asymmetric nanomaterials.

The second one is to further understand the growth process and formation mechanism of asymmetric nanomaterials. Limited by the current characterization methods and synthesis techniques, although some consensus has been reached, many assembly mechanisms remain unclear. Therefore, it is necessary to comprehensively observe the growth process of materials by means of in situ characterization techniques, such as in situ electron microscopy, cryo-electron microscopy, synchrotron radiation, and other advanced characterization methods. In turn, a deep understanding of the formation mechanism will help us better regulate the structures of materials in a controlled manner. Additionally, with the rapid development of computer science, some new structures or new methods can be predicted through theoretical simulations and calculations, which can provide a theoretical guidance for designing of some new and unique asymmetric structures.

Third, the function-oriented preparation should be the focus of future research to maximize the performances of asymmetric nanomaterials. Through the establishment of theoretical models, the internal relation between the structure and performance should be further explored by systematically investigating the influence of particle size, composition, configuration and other factors on their performance, which would better serve the design of the asymmetric structures. On the other hand, designing customized asymmetric structures to meet specific application requirements is of importance. For example, for the application of energy storage and conversion, it usually requires that the structure possesses larger effective surface area, higher packing density, better electrical conductivity, and excellent mechanical stability. In terms of biomedical application, it is necessary to fabricate asymmetric structure with small size, good biocompatibility, and easily functional inside and outside surfaces. The last but not least is to design asymmetric structures with more complex structures and components, thereby extending their application prospects. That needs the researchers in various fields to merge their expertise and cooperate together to explore new application opportunities.
